# Job Satisfaction among Care Aides in Residential Long-Term Care: A Systematic Review of Contributing Factors, Both Individual and Organizational

**DOI:** 10.1155/2015/157924

**Published:** 2015-08-05

**Authors:** Janet E. Squires, Matthias Hoben, Stefanie Linklater, Heather L. Carleton, Nicole Graham, Carole A. Estabrooks

**Affiliations:** ^1^School of Nursing, University of Ottawa, 451 Smyth Road, Ottawa, ON, Canada K1H 8M5; ^2^Ottawa Hospital Research Institute, Centre for Practice-Changing Research (CPCR), 501 Smyth Road, Room 1282, Box 711, Ottawa, ON, Canada K1H 8L6; ^3^Faculty of Nursing, University of Alberta, Level 3, Edmonton Clinic Health Academy, 11405 87 Avenue NW Edmonton, AB, Canada T6G 1C9

## Abstract

Despite an increasing literature on professional nurses' job satisfaction, job satisfaction by nonprofessional nursing care providers and, in particular, in residential long-term care facilities, is sparsely described. The purpose of this study was to systematically review the evidence on which factors (individual and organizational) are associated with job satisfaction among care aides, nurse aides, and nursing assistants, who provide the majority of direct resident care, in residential long-term care facilities. Nine online databases were searched. Two authors independently screened, and extracted data and assessed the included publications for methodological quality. Decision rules were developed a priori to draw conclusions on which factors are important to care aide job satisfaction. Forty-two publications were included. Individual factors found to be important were empowerment and autonomy. Six additional individual factors were found to be *not* important: age, ethnicity, gender, education level, attending specialized training, and years of experience. Organizational factors found to be important were facility resources and workload. Two additional factors were found to be *not* important: satisfaction with salary/benefits and job performance. Factors important to care aide job satisfaction differ from those reported among hospital nurses, supporting the need for different strategies to improve care aide job satisfaction in residential long-term care.

## 1. Background

### 1.1. Aging and Residential Long-Term Care

In the first half of the 21st century, the global population 60 years or over is projected to expand threefold to nearly 2 billion, with 33 countries having more than 10 million people 60 years of age or over [1]. With this dramatic demographic shift come sharp increases in numbers of older adults with age-related dementias (ARDs) [2–4]. ARDs are a world-wide public health concern, with nearly 7.7 million new cases globally each year [5]. ARDs are the main factor escalating the need for residential long-term care (LTC) [6–8]. Without dramatic breakthroughs in ARD prevention, treatment, or management, the need for residential LTC facilities will increase as the population ages.

Residential LTC facilities offer 24-hour on-site housing and health care services to the elderly, defined as persons of age 65 and older. The individuals cared for at these facilities are frail, vulnerable, functionally dependent older adults who frequently suffer from a range of chronic diseases or disabilities [9, 10]. There are varying terms to describe residential LTC facilities, for example, residential care, assisted living facilities, nursing homes, long-term care homes, and residential aged care. These facilities offer different levels of care and may be individual structures or associated within or with community care centres or hospitals.

Nonprofessional workers (care aides, also commonly referred to as personal care workers, nursing assistants, and nurse aides) provide most direct nursing care in these facilities. In Canada and the USA, these individuals provide 70–80% of direct care to residents in residential LTC facilities [11–15]. These workers often lack adequate formal qualifications [16], continuing education, and monitoring [16], which poses a serious concern in meeting minimum standards of care in LTC [17]. Care aide duties may include apparently simple tasks related to personal hygiene, toileting, feeding, and housekeeping, but aides are also vital to the quality of life of our growing vulnerable older adult population.

Across all care settings, we currently see widespread shortages of all levels of nursing care providers and high turnover rates. This global issue is increasingly important to both developed and developing countries [18–21] and of increasing concern in many countries [17, 18] and the LTC sector. Staff turnover in residential long-term care facilities ranges from 40% to 500% [22, 23]. Numerous factors have been linked to turnover of nursing care providers; job satisfaction however is by far the most frequently cited [24–26].

### 1.2. Job Satisfaction

Multiple definitions of job satisfaction abound in the literature. For this review, we defined job satisfaction using the traditional model frequently cited in empirical studies of job satisfaction of nursing care providers. This model focuses on job satisfaction as the affective orientation of an employee towards his or her work (i.e., on the feelings an individual has about his or her job [20, 27]). This “affective” focus can be seen in frequently cited definitions of job satisfaction scholars such as Locke [28, 29] who describes job satisfaction as a “pleasurable or positive emotional state resulting from the appraisal of one's job or job experiences” and Brooke and colleagues who describe job satisfaction as “an affective response to the job situation” [30].

Not all dissatisfied staff will leave their job, but dissatisfaction may impact their work, their coworkers, and the quality of resident care delivered. Dissatisfied staff often show signs of an unreliable work ethic, such as tardiness and taking unscheduled days off [31]. Some dissatisfied staff show greater aggression towards other workers [32] and residents [33]. Job dissatisfaction is associated with reduced quality of resident care [34] and resident quality of life [35], as well as reduced ability of organizations to change [36]. Conversely, caregivers (including care aides) who report perceiving high quality of care in their facilities also report higher satisfaction with their job [31]. For the last several decades, quality of care in some residential LTC facilities has been consistently reported as substandard [37–39]. This fact, coupled with evidence of residential LTC facilities' limited ability to change in a meaningful way [31], highlights the importance of understanding job satisfaction of care aides in these facilities.

While multiple individual studies examine factors related to care aide's job satisfaction and/or job satisfaction in residential LTC facilities, this evidence has not yet been synthesized. However, a synthesis of factors associated with job satisfaction among hospital registered nurses was recently published. In that review, Lu and colleagues [20] found that job satisfaction is closely related to working conditions and the organizational environment, job stress, role conflict and ambiguity, role perception and role content, and organizational and professional commitment [20]. The* purpose* of this systematic review is to synthesize the evidence on factors (both individual and organizational) associated with job satisfaction among care aides in residential LTC facilities.

## 2. Methods

### 2.1. Selection Criteria for Types of Studies

Primary studies that used experimental (randomized controlled trials, clinical trials, and quasi-experimental, e.g., pre/posttest [40]) and nonexperimental (observational and qualitative [40]) designs examining factors associated with job satisfaction for care aides in residential LTC facilities were eligible for inclusion. Studies were limited to those published in English, with no restrictions on country of origin or publication date.

### 2.2. Selection Criteria for Types of Participants, Factors, and Outcomes

Participants included care aides, nurse aides, and/or nursing assistants.* Care aide* was defined as a nonprofessional worker providing direct resident care, under the supervision of a registered nurse (RN) or licensed practical nurse (LPN) or registered practical nurse (RPN).* Nursing assistant/aide* (NA) was defined as a person who has completed a brief health care training program and who provides support services for RNs and LPNs/RPNs. An NA is termed a certified nurse aide (CNA) when certified by a state agency (USA) or province (Canada) [41]. Factors (independent variables) of interest were any individual or organizational variable associated with job satisfaction, the outcome of interest. We defined job satisfaction as the affective orientation of an employee towards his or her work (i.e., on the feelings an individual has about his or her job [20, 27]). We included studies of job satisfaction that met this definition. We included studies of job satisfaction in other care providers only if a separate analysis of care aide/NA job satisfaction was provided or could be extracted. Only studies published in English were eligible for inclusion.

### 2.3. Search Strategy for Identification of Studies

The search strategy (Table 1) was developed in consultation with a health sciences librarian. We searched nine online databases: the Cochrane Database of Systematic Reviews, CINAHL, Business Source Complete, Medline, EMBASE, AARP AgeLine, Web of Science, SCOPUS, and ABI Inform. Key words included long-term care, care aide, and job satisfaction (and their synonyms).

### 2.4. Study Identification

Two team members independently screened all abstracts identified by the search strategy (*n* = 967 after removal of duplicates). Full text copies were retrieved for all citations identified as potentially relevant to our review aim or with insufficient information to make a decision on relevance (*n* = 164). Any article not meeting all inclusion criteria outlined above was excluded from the review.

**Figure 1:**
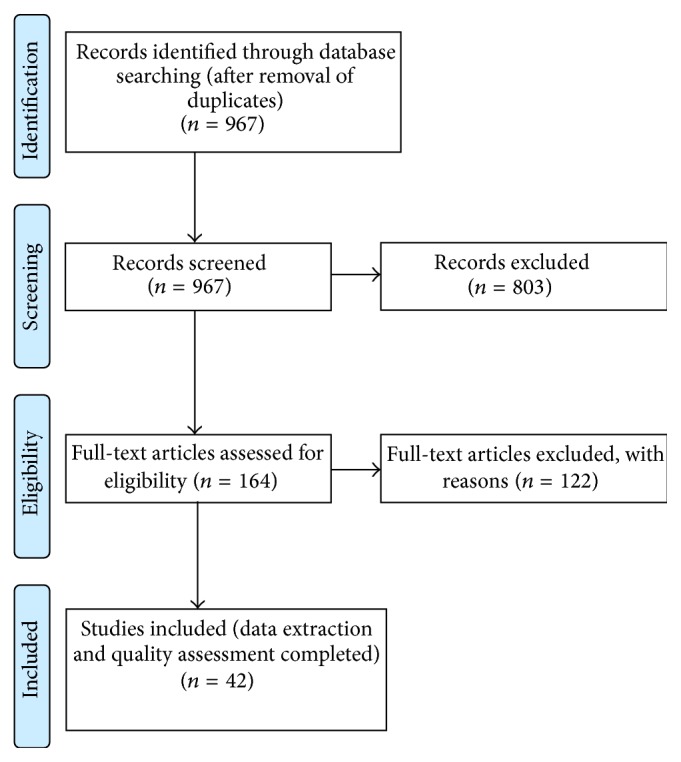
PRISMA flow diagram.

Two reviewers independently assessed all retrieved articles; 42 articles were retained (see PRISMA flow diagram in Figure 1). Screening discrepancies were resolved through consensus.

### 2.5. Quality Assessment

Methodological quality of the final set of included articles was independently assessed by two reviewers with disagreements resolved through consensus. Four previously validated assessment tools were used. Quantitative studies were assessed using 1 of 3 tools: (1) the Quality Assessment and Validity Tool for Cross-Sectional Studies, (2) the Quality Assessment and Validity Tool for Pre/Posttest Studies, and (3) the Quality Assessment Tool for Quantitative Studies (used for randomized controlled trials). The original tools are described in detail in previously published systematic reviews (e.g., [42–45]). Quality assessment considered appropriateness of study design based on the research objectives, sample, measurement of key variables (individual and organizational factors) and the outcome of interest (job satisfaction), and appropriateness of the statistical analysis.

The first two tools, the* Quality Assessment and Validity Tool for Cross-Sectional Studies* and the* Quality Assessment and Validity Tool for Pre/Posttest Studies,* were originally developed by members of our team based on Cochrane guidelines (in existence since 2001) and the medical literature [86, 87] and have been used in other published systematic reviews by our group [42–45]. The cross-sectional tool contains a maximum of 16 points and assesses studies in three core areas: sampling, measurement, and statistical analysis. The pre/posttest tool contains a maximum of 18 points and assesses studies in 6 core areas: sampling, design, control of confounders, data collection and outcome measurement, statistical analysis, and dropout. To derive a final quality score for each article, we divided the total points scored by the total points possible (16 or 18 minus the number of points not applicable for the article). Each study was then classified as weak (≤0.50), moderate-weak (0.51 to 0.65), moderate-strong (0.66 to 0.79), or strong (≥0.80). This rating system has been used in several recent reviews [43–45] and is based on a scoring system developed by De Vet et al. [88]. These two tools were used to assess the methodological quality of all cross-sectional (*n* = 29) and pre/post (*n* = 7) studies included in our review.

The third quality assessment tool used in this review was the* Quality Assessment Tool for Quantitative Studies*, developed by the Effective Public Health Practice Project, Canada. This tool has been judged suitable to be used in systematic reviews of effectiveness (measuring interventions) [89] and been shown to have content and construct validity [90]. The tool assesses studies on the basis of six areas: selection bias, study design, confounders, blinding, data collection methods, and withdrawals/dropouts. Each article is scored as weak, moderate, or strong in each of these areas according to preset criteria within the tool. The tool developers do not provide a means for calculating an overall quality score. However, in order to compare the quality scores for the included articles assessed with this tool to those that used cross-sectional and pre/posttest tools, we derived an overall quality score. We applied the scoring system of this tool used in a previously published review [44]. This score was derived by assigning values of 1, 2, and 3 to the categorizations of weak, moderate, and strong respectively. A final quality score was then obtained by dividing the summative score obtained by the total amount of points possible. Each study was classified as weak (1 to 1.5), moderate-weak (1.6 to 2.0), moderate-strong (2.1 to 2.5), or strong (>2.5) by applying the same categorization system used (and published) in the cross-sectional and pre/posttest tools. The* Quality Assessment Tool for Quantitative Studies Tool* was used to assess RCT studies included in this review (*n* = 1).

Qualitative studies were assessed using the* Critical Appraisal Skills Programme (CASP) Quality Assessment Tool *[91]. This tool assesses qualitative studies through 10 questions on research aims, appropriateness of research design, appropriateness of recruitment strategy, data collection, relationship between researcher and participants, ethical issues, data analysis, statement of findings, and value of the research [91]. A final quality score for each article was then obtained by dividing the summative score obtained by the total amount of points possible. Each study was classified using the same rating scale as for the cross-sectional and pre/posttest studies: weak (≤0.50), moderate-weak (0.51 to 0.65), moderate-strong (0.66 to 0.79), or strong (≥0.80).

### 2.6. Data Extraction and Synthesis

One team member extracted data from all included articles, double-checked by a second team member for accuracy. Discrepancies in data extraction were resolved through consensus. Data were extracted on year of publication, title, journal, country of origin, purpose/objectives, data collection methods, study design, sample size and setting, job satisfaction measure (including number of items, reliability, and validity), independent variables investigated (individual and organizational factors), analyses, and main outcome(s).

Data on individual factors were grouped into five broad categories (each having subgroups). The five broad categories were (1) sociodemographic, (2) education, (3) healthcare provider characteristics, (4) personal life, and (5) other. Data on organizational factors were also grouped into five categories, again with subgroups. The five broad organizational categories were (1) facility, (2) work environment, (3) supervision, (4) staffing, and (5) other. Categories (and their subgroups) were not predetermined; after reviewing and extracting data, we found that factors relevant to our aim centered on these themes. We used the primary studies authors' conceptualizations in this grouping. For example, if an author reported investigating autonomy, it was classified as autonomy in our synthesis; we did not reclassify any variables based on the definitions provided in the primary studies. Grouping the factors facilitated comparing and interpreting their importance to care aide job satisfaction in residential LTC facilities.

We used a vote-counting approach to synthesize the quantitative evidence. The overall assessment of a relationship between a factor and job satisfaction was based on the percentage of studies demonstrating, or failing to demonstrate, statistically significant associations. As recommended by Grimshaw et al. [92], we supplemented this by extracting direction and magnitude of effect for all factors displaying statistically significant effects (*p* < 0.05), where provided. If a study included multiple analyses (e.g., univariate, bivariate, and/or multivariate), we relied on the highest level model (e.g., multivariate where available).

Qualitative findings were assessed for themes and summarized narratively. We applied the following previously published* a priori* rules [45] to guide our quantitative synthesis.

1. To conclude whether or not a factor (individual or organizational) was associated with job satisfaction, it had to be assessed four or more times (this could reflect two assessments of different variables comprising the same factor from one study). If a factor was assessed fewer than 4 times it was coded as inconsistent (i.e., insufficient evidence to reach a conclusion).
2. Factors assessed four or more times were coded as
  - significant with (important to) job satisfaction if 60% or more of the quantitative tests showed a significant association between the factor and job satisfaction;
  - nonsignificant with (not important to) job satisfaction if 60% or more of the quantitative tests showed a nonsignificant association between the factor and job satisfaction;
  - equivocal with (undetermined importance to) job satisfaction if <60% of the quantitative tests showed significant/nonsignificant associations between the factor and job satisfaction.

## 3. Results

### 3.1. Description of Studies

Forty-two studies were included in the review. The majority (*n* = 29) of studies used a cross-sectional survey design [9, 10, 33, 46–48, 52–54, 56–58, 60–62, 64, 66–71, 73–75, 77–79, 93]. Of these, 1 study used mixed methods (survey plus qualitative data) [78], 1 study used a randomized controlled trial [59], and 7 studies used observational before-and-after quasi-experimental (pre/posttest) design [49, 51, 55, 65, 72, 76, 80]. One of the quasi-experimental studies also used mixed methods and included qualitative data [55]. Five additional studies used a qualitative design [81–85]. Overall, our sample included 37 studies with quantitative statistical data and 7 studies with qualitative data.

Studies were conducted with CNAs (*n* = 24), NAs (*n* = 7), and care aides (*n* = 5); 6 studies included multiple groups. Studies were set in residential LTC facilities (*n* = 22), other LTC facilities (*n* = 7), assisted living facilities (*n* = 1), skilled nursing facilities (*n* = 2), and combined assisted living/skilled nursing facilities (*n* = 1). Countries of origin were the USA (*n* = 37), Taiwan (*n* = 2), Sweden (*n* = 1), Canada (*n* = 1), and Australia (*n* = 1). Studies were published between 1976 and 2012 with the majority being published after 2000 (*n* = 28). Different measures of job satisfaction were used across the studies. Only 4 job satisfaction tools were used in greater than one study: Minnesota Satisfaction Questionnaire (*n* = 4 studies), Job Descriptive Index (*n* = 3 studies), Benjamin Rose Institute Job Satisfaction Scale (*n* = 3 studies), Job Diagnostic Survey (*n* = 3 studies), and Job Attitude Scale (*n* = 2 studies). Details on included studies are given in Table 2; a list of studies was excluded and the reason(s) for their exclusion are in Additional File 1 (in Supplementary Material available online at http://dx.doi.org/10.1155/2015/157924).

### 3.2. Methodological Quality of Included Studies

We completed 44 quality assessments on the 42 included studies; the 2 studies [55, 78] with mixed methods designs both had 2 quality assessments done. Details of methodological quality assessments of all 42 studies are in Additional File 2.

From the 44 quality assessments, 5 (11%) studies were rated strong [9, 78, 81, 84, 85], 6 (14%) high moderate [53, 56, 66–68, 73], 15 (34%) low moderate [10, 52, 57, 58, 61, 64, 65, 69, 71, 72, 75, 76, 78, 83, 93], and 18 (41%) weak [33, 46–49, 51, 54, 55, 59, 60, 62, 70, 74, 77, 79, 80, 82]. Differences in quality assessment arose mainly from sample representativeness, treatment of missing data, and appropriateness of statistical test(s) used.

We conducted a sensitivity analysis, comparing findings from all studies with those rated moderate and strong. No significant differences were noted; thus, we report findings from all studies.

### 3.3. Individual/Organizational Factors and Job Satisfaction

#### 3.3.1. Quantitative Findings

A total of 33 and 25 studies investigated the statistical association of care aide job satisfaction with individual and organizational factors, respectively. Details of the statistical effects including direction of effect and significance of the studies meeting our criteria to be able to draw a conclusion (i.e., assessed four or more times) are presented in Table 3 (individual factors) and Table 4 (organizational factors). Additionally, an overall picture of the findings and the resulting conclusions drawn are depicted in Table 5 (individual factor conclusions) and Table 6 (organizational factor conclusions). A summary of findings with respect to the relationship between job satisfaction and individual and organizational factors that were assessed less than four times can be found in Additional File 3.

As illustrated in Tables 3 and 5, 11 individual (care aide) factors spanning 4 of the 5 main categories were assessed 4 or more times. Two of these factors, both under the category of healthcare provider characteristics, had a significant positive relationship with care aide job satisfaction: empowerment and autonomy. Six additional individual factors (spanning 3 categories; categories are identified in brackets) showed no relationship to job satisfaction: age (sociodemographics), ethnicity (sociodemographics), gender (sociodemographics), level of education/years of education (education), special training (education), and years of experience as a care aide (healthcare provider characteristics). The remaining 3 individual factors assessed 4 or more times showed equivocal findings in relation to care aide job satisfaction: current position (personal characteristics), employment status (personal characteristics), and stress (personal life).

Tables 4 and 6 depict the five organizational factors that were assessed 4 or more times; these 5 factors spanned 3 organizational categories. Two of these factors had a significant positive relationship overall with care aide job satisfaction (the categories are identified in brackets): resources (facility) and workload (workload). Two factors showed no relationship to job satisfaction: satisfaction with salary/benefits (work environment) and job performance (work environment). The remaining organizational factor assessed 4 or more times was support from coworkers (work environment) which had an equivocal relationship with care aide job satisfaction.

#### 3.3.2. Qualitative Findings

Most factors identified in the qualitative data were organizational in nature and were reported in a single study (Table 7). Overall, qualitative findings support the conclusions drawn from the synthesis of the quantitative data. Factors related to work environment were most frequently mentioned in both quantitative and qualitative studies; respondents in all 7 qualitative studies discussed 1 or more work environment factors. Of particular significance is that 3 factors not studied quantitatively emerged in the qualitative studies as important to care aide job satisfaction:* contact/relationships with residents *[82–85],* nature of the job* (care aide work) [62, 84, 85], and* opportunity for learning and advancement *[62, 74, 82].

## 4. Discussion

### 4.1. Summary of Findings

This systematic review examined the evidence on associations between individual and organizational factors and care aide job satisfaction. The body of evidence provides significant empirical support for the relationship of several factors to an increase in care aides' job satisfaction. Important individual factors identified were* empowerment *and* autonomy*. Six individual factors were shown to be* not *important: age, ethnicity, gender, education level, attending specialized training, and years of experience. Important organizational factors were* facility resources* and* workload*. Two organizational factors were found to be* not* important: care aide satisfaction with salary/benefits and job performance.

### 4.2. Comparison with the Review on Job Satisfaction among Hospital Registered Nurses

No previous syntheses exist on job satisfaction in care aides or with nursing care providers in residential LTC, but job satisfaction among registered nurses in hospitals was subject to a recent systematic review [20]. Both studies found the individual factors autonomy and empowerment to be important to job satisfaction. However, several important differences between our review and the hospital registered nurse review are evident. First, in the hospital registered nurse group, job satisfaction was closely related to working conditions and organizational and environmental factors, namely, job stress, role conflict/ambiguity, role perception/content, organizational commitment, and professional commitment. While we found similar overall categories, we found different factors within these categories to be important to care aide job satisfaction in residential LTC. For example, both nurse job satisfaction and care aide job satisfaction were closely related to working conditions, but care aides noted workloads and availability of facility level resources as important (Table 4) while registered hospital nurses noted team cohesiveness and physical conditions of the unit to be important [20]. Coworker support had a high moderate relationship to hospital registered nurse job satisfaction [20] but was only equivocally related to care aide job satisfaction (Table 4). Second, while age, years of experience, and education level all had significant relationships with job satisfaction in hospital registered nurses [20], these individual factors were not consistently significant to care aide job satisfaction (Table 3). Third, stress had a strong relationship with registered nurse job satisfaction [20] but only an equivocal relationship for care aides (Table 3). Each of these discrepancies may reflect true differences between groups (i.e., between registered nurses and care aides) and/or settings (i.e., between hospitals and residential LTC) or may reflect differences in synthesis methods. Lu and colleagues [20] reported all factors displaying statistically significant findings in any study as important to registered nurse job satisfaction. In this synthesis, we applied stringent decision rules. To classify a factor as important to job satisfaction, we required it to be tested 4 or more times and have significant findings in at least 60% of those studies. Regardless of the reason(s) for differences between the two reviews, these differences highlight the importance of conducting systematic reviews in LTC and with nursing care provider groups other than registered nurses. These findings also importantly suggest that different strategies may be needed to improve care aide job satisfaction in residential LTC facilities compared to hospital nurses.

### 4.3. Methodological Implications for Future Research

Systematic reviews typically identify problems with internal validity of research under investigation. Future studies on factors related to care aide job satisfaction need to emphasize methodological quality, to reduce bias and increase confidence in this growing body of knowledge. Researchers will then be able to design better-informed interventions to improve care aide job satisfaction, recruitment, and retention of this vital staffing group.

Two important methodological limitations of the studies conducted to date included in this review are methodological quality and statistical rigor. Few studies included in this review were of high moderate or strong methodological quality, illustrating a clear need for well-designed, robust studies in the area. Studies also varied in statistical rigor, although we observed a promising trend in recent studies to more robust analyses (multivariate regression over bivariate and univariate statistics). Given the heterogeneity among studies, however, we could only draw conclusions on which factors are associated with job satisfaction and not on which factors predict job satisfaction. Future research should look more closely at prediction; only 14 (38%) of our 37 included quantitative studies reported prediction (multivariate regression).

### 4.4. Limitations of This Review

While we used rigorous methods in this review, there are limitations. First, we did not search all grey literature databases; therefore, this review may not include all relevant work. Second, we did not attempt to clarify unclear study details by contacting the study authors; nonreported aspects of methods may have lowered scores in our quality assessment. Third, we used vote counting to synthesize quantitative data. Vote counting does not account for effect sizes (it gives equal weight to all associations irrespective of magnitude) or precision of estimates (it gives equal weight to comparisons irrespective of sample size). To lessen these problems we reported the number of comparisons showing statistically significant effects (regardless of direction) and the magnitude of effect for significant findings [92]. Fourth, there is a small possibility of a culture effect given the fact that different countries may experience job satisfaction differently and also have different determinants to job satisfaction. This effect should however be minimal given the fact that the vast majority of studies identified are from the USA (*n* = 37 of 42). Finally, our criteria for reaching a conclusion on the factors important to job satisfaction were stringent and while we considered overall methodological quality of the included studies in determining these conclusions, we did not take into account specific individual methodological strengths and weaknesses of each study in determining which factors were important overall to care aide job satisfaction.

## 5. Conclusions

We identified several factors as important to care aide job satisfaction. Individual factors were* empowerment* and* autonomy*; organizational factors were* facility resources* and* workload*. Equally important, several factors were shown to be* not* important: age, ethnicity, gender, education level, attending specialized training, years of experience, satisfaction with salary/benefits, and job performance. Factors identified as important hold promise as targets of care aide job satisfaction interventions. However, methodological problems inherent in many studies suggest that additional research using more robust study designs and multivariate assessment methods is required. Future research might also usefully test the association between care aide job satisfaction and the factors identified in qualitative studies included in this review:* contact/relationships with residents*,* nature of the job* (care aide work), and* opportunity for learning and advancement*.

## Supplementary Material

Additional file 1 contains a list of studies excluded from the review at the stage of full-text screening and the reasons for their exclusion.
Additional file 2 contains the details of the methodological quality assessment conducted on all 42 studies included in the review. Additional file 3 provides a summary of findings with respect to the relationship between job satisfaction and individual and organizational factors that were assessed less than 4 times. Factors assessed 4 or more times are presented in the body of the paper.

## Figures and Tables

**Table 1:** Search strategy (all searches performed through to May 1, 2013).

Database	Search terms
CINAHL	(MH “Nursing Assistants”) OR (MH “Nursing Home Personnel”) “health care aide^*∗*^” or “nursing assistant^*∗*^” or “nurs^*∗*^ aide^*∗*^” or “personal care aide^*∗*^” or “resident companion^*∗*^” or “geriatric aide^*∗*^” or hca (MH “Job Satisfaction”) (“Job satisfaction”) or career N2 satisf^*∗*^ or work N2 satisf^*∗∗*^ or employ^*∗*^ N2 satisf^*∗*^

Business Source Complete	(“health care aide^*∗*^” or “nursing assistant^*∗*^” or aide^*∗*^ or “nurs^*∗*^ aide^*∗*^” or “personal care aide^*∗*^” or “resident companion^*∗*^” or “geriatric aide^*∗*^”) and (“job satisfaction” or satisf^*∗*^ N2 work^*∗*^ or satisf^*∗*^ N2 employ^*∗*^ satisf^*∗*^ N2 career^*∗*^)

Medline	Nurses' Aides/(health care aide^*∗*^ or health care attendant^*∗*^ or HCA or personal care or personal care attendant or nursing assistant^*∗*^ or resident companion or geriatric aide^*∗*^).tw. ((auxiliary adj 1 nurs^*∗*^) or (nurs^*∗*^ adj 1 aide^*∗*^)).tw. Job Satisfaction/job satisfaction.tw. (satisf^*∗*^ adj 2 (work^*∗*^ or employ^*∗*^ or career^*∗*^)).tw.

EMBASE	nursing assistant/(health care aide^*∗*^ or health care attendant^*∗*^ or HCA or personal care or personal care attendant or nursing assistant^*∗*^ or resident companion or geriatric aide^*∗*^).tw. ((auxiliary adj 1 nurs^*∗*^) or (nurs^*∗*^ adj 1 aide^*∗*^)).tw. job satisfaction/job satisfaction.tw. (satisf^*∗*^ adj 2 (work^*∗*^ or employ^*∗*^ or career^*∗*^)).tw.

AARP Ageline	“Nurses-Aides”.de. (health care aide^*∗*^ or health care attendant^*∗*^ or HCA or personal care or personal care attendant or nursing assistant^*∗*^ or resident companion or geriatric aide^*∗*^).tw. ((auxiliary adj 1 nurs^*∗*^) or (nurs^*∗*^ adj 1 aide^*∗*^)).tw. “Job-Satisfaction”.de. job satisfaction.tw. (satisf^*∗*^ adj 2 (work^*∗*^ or employ^*∗*^ or career^*∗*^)).tw.

Web of Science	TS = (“health care aide^*∗*^” or “health care attendant^*∗*^” or HCA or “personal care attendant^*∗*^” or “nursing assistant^*∗*^” or “resident companion^*∗*^” or “geriatric aide^*∗*^”) AND TS = (“job satisfaction”) Databases = SCI-EXPANDED, SSCI, CPCI-S

SCOPUS	(TITLE-ABS-KEY(“health care aide^*∗*^” OR “health care attendant^*∗*^” OR hca OR “personal care attendant^*∗*^” OR “nursing assistant^*∗*^” OR ”resident companion^*∗*^” OR “geriatric aide^*∗*^”) AND TITLE-ABS-KEY(“job satisfaction”))

ABI Inform	(“health care aide^*∗*^” OR “nursing assistant^*∗*^” OR “nurs^*∗*^ aide^*∗*^” OR “personal care aide^*∗*^” OR “resident companion^*∗*^” OR “geriatric aide^*∗*^”) AND (“job satisfaction”)

Cochrane	job satisfaction

**Table 2:** Characteristics of included studies.

First author, *journal* (year)	Study design	Location/sample/subjects	Data collection method	Explanatory variables studied (individual variables)	Job satisfaction instrument			Quality
					Job satisfaction measure(s)	Reliability	Validity	
*Quantitative studies (n = 37) *								

Albanese [46], *Thesis* (1995)	Cross-sectional (single group)	*Country*: USA *Sample size*: *n = *255 *Subjects*: CNAs employed in 14 residential care facilities	Questionnaire	*Social organizational variables*: number of beds, occupancy rate, immediate supervisor status, NA employment status, CNA overtime hours, NA night shift, nursing director length of employment, nursing director professional membership, administrator education, proportion of Medicaid residents, proportion of Medicare residents, number of children living with NA, CNA satisfaction with religious life *Work role relations variables*: positive and negative resident relations, positive and negative supervisor relations, positive and negative visitor relations *CNA job stress *	Quinn and Staines Job Satisfaction Scale	*α* = 0.76	Not reported	Weak

Allensworth-Davies [47], *Health Care Management Review* (2007)	Cross-sectional survey (single group)	*Country*: USA. *Sample size*: *n = *135 *Subjects*: NAs at 4 new England's residential care facilities	Questionnaire	(i) Workplace cultural competency (ii) Age (iii) Racioethnicity (iv) Autonomy	General satisfaction scale from the Job Diagnostics Survey (5 items)	Not reported	Not reported	Weak

Berg [48], *Scandinavian Journal of Rehabilitation Medicine* (1976)	Cross-sectional survey (single group)	*Country*: Sweden *Sample size*: *n = *233 *Subjects*: CNAs in one geriatric LTC hospital (20 wards)	Questionnaire	(i) Determinants for the mean scores of the seven scales (one of them “satisfaction with the work itself”): age, length of employment, and training course passed yes/no (ii) Determinants for the overall JS question (the seven questionnaire scales): (1) satisfaction with the work itself, (2) perceived strain, (3) adjustment to geriatric work, (4) relation with colleagues and supervisors, (5) perceived need for info., (6) perceived demand for physical and psychic strength, and (7) perceived need for education	53 items (7 scales) in the entire questionnaire; 4 items in one scale were related to JS	Not reported	Not reported	Weak

Blackmon [49], *Thesis* (1993)	Before-and-after^*∗∗*^	*Country*: USA. *Sample size*: *n = *188 (sample size reduced to 88 in regression due to application of the listwise procedure) *Subjects*: CNAs (number of residential care facilities not reported)	Questionnaire	(i) Intervention: training (ii) Regression: tested knowledge of how to perform care tasks, perceived knowledge of how to perform care tasks, sex, age, education, length of employment, race, and degree of religiosity	Each of the 3 items were borrowed from the JS scale developed by Kahn (1964) [50]	Not reported	Not reported	Weak

Braun [51], *Journal of Elder Abuse and Neglect* (1997)	Before-and-after^*∗∗*^	*Country*: USA *Sample size*: *n = *105 *Subjects*: CNAs (number of NHs not reported)	Questionnaire	Elder abuse and neglect prevention training (locally developed program consisting of videos, booklet, and interactive workshop)	Asked to rate their level of JS on a scale from 1 to 10	Not reported	Not reported	Weak

Burgio [52], *The Gerontologist* (2004)	Cross sectional (between groups quasi-comparison design)	*Country*: USA *Sample size*: *n = *178 *Subjects*: CNAs from 4 NHs	(i) Direct structured observation (ii) Structured questionnaires (iii)Analysis of resident records	(i) Permanent versus rotating shift assignment (ii) Isolated and combined effects of work shift	Job Satisfaction Index (JSI)	*α* = 0.69–0.89 in a previous study	Not reported	Low moderate

Choi [53], *Research in Nursing and Health* (2012)	Secondary analysis of cross-sectional survey data	*Country*: USA *Sample size*: *n = *2,254 *Subjects*: CNAs within 516 NHs	Computer-assisted telephone interviewing (CATI) system where interviewers asked questions over the telephone (Data from the existing National Nursing Assistant Survey and National Nursing Home Survey)	*Fixed effects work-related factors* *Level 1 (individual CNA) * ** **Supportive supervision, perception of being valued, work-related injury, hourly wage, employee benefits, health insurance *Level 2* (residential care facilities) Bed sizes, for-profit/nonprofit, location (metropolitan, micropolitan, rural), percent of Medicare residents, percent of Medicaid residents, RN HPPD, LPN HPPD, CNA HPPD *Personal factors * ** ** Age (years), white/nonwhite, education level (high school or less), number of jobs in the past 5 years (0–5+)	A single-item measure for an overall measure of JS. The item was scored using a 4-point Likert-type scale, ranging from 1 (extremely dissatisfied) to 4 (extremely satisfied)	Not reported	Not reported	High moderate

Cready [54], *Journal of Gerontological Nursing* (2008)	Cross-sectional (single group)	*Country*: USA *Sample size*: *n = *434 *Subjects*: HCAs and nurses from 10 NHs	Questionnaire	Empowerment (low, medium, or high)	Not reported—authors stated that “when available, items were taken from previous studies [55]”	Not reported	Not reported	Weak

Friedman [56], *The Gerontologist* (1999)	Cross-sectional (two-group comparison; quasi-experimental) survey	*Country*: USA *Sample size*: *n = *349 *Subjects*: CNAs in 10 NHs (5 PACE, 5 non-PACE)	Questionnaire	(i) Demographics (age, education, experience with elderly in childhood) (ii) Job description (iii) Working in PACE versus regular residential care facilities	(i) Minnesota Satisfaction Questionnaire (ii) Two questions rated on a scale from 1–5 on: (iii) “how satisfied they were with their current job” (iv) “how likely they were to leave their job in the next year”	*α* = 0.90	Stated validity in previous studies	High moderate

Garland [57], *Journal of Aging Studies * (1989)	Cross-sectional survey (single group)	*Country*: USA *Sample size*: *n = *138 *Subjects*: NAs from 45 NHs	Questionnaire	Fifteen items broken down into four groups: (i) Supervision (having necessary supplies; enough time; amount of work manageable; access to necessary info; knowing how supervisor is evaluating you; not knowing what supervisor expects; being sure of what supervisor wants; conflicting orders from people in authority) (ii) Personal recognition (supervisor asks for your opinion; others care how well you do your job) (iii) Family/work conflict (job interferes with family life; family life interferes with job) (iv) Qualifications (wish for more training; feel qualified)	Modification of Kahn et al. (1964) [50] Job Satisfaction Scale	*α* = 0.74	Not reported	Low moderate

Gittell [58], *Human Resource Management Journal* (2008)	Cross-sectional survey (single group)	*Country*: USA *Sample size*: *n = *252 *Subjects*: CNAs from 2 specific units at 15 different LTC facilities (10 nonprofit and 5 for-profit)	Questionnaire	(i) Demographics (ii) Facility characteristics (size and ownership) (iii) Relational coordination (communication and relationships)	One JS item “overall, how satisfied are you with your job?”	Not reported	Not reported	Low moderate

Goldwasser [59], *Journal of Mental Health and Aging * (1996)	RCT (with four groups)	*Country*: USA *Sample size*: *n = *27 *Subjects*: CNAs in one LTC facility	Questionnaire	(i) Model of care (reminiscence versus present focused) (ii) Present during resident interviews versus not present during interviews	Short form of the Minnesota Satisfaction Questionnaire (20 items)	Internal consistency coefficients of the subscales range from 0.80 s to 0.90 s	Not reported	Weak

Grieshaber [60], *The Health Care Supervisor* (1995)	Cross-sectional survey design (2 groups)	*Country*: USA *Sample size*: *n = *79 *Subjects*: CNAs	Questionnaire	(i) Facility type (urban versus suburban) (ii) Age (iii) Education (iv) Job tenure (v) Occupation tenure	Short form of the Minnesota Satisfaction Questionnaire	Reliable in other studies, but no numbers were reported	Stated valid in other studies	Weak

Gruss [61], *Thesis* (2007)	Cross-sectional survey (single group)	*Country*: USA *Sample size*: *n = *42 *Subjects*: CNAs from 3 dementia care units in 3 LTC facilities	Questionnaire	IV = empowerment: (i) Structural empowerment (summary score of 4 subscales: opportunity, information, support, resources) (ii) Psychological empowerment	Abridged Job Description Index (25 items)	Not indicated for this sample, referred to other studies without reporting numbers	Not indicated for this sample, referred to other studies	Low moderate

Holtz [62], *Journal of Gerontological Nursing* (1982)	Cross-sectional survey (single group)	*Country*: USA *Sample size*: *n = *31 *Subjects*: HCAs from 3 level II and III residential care facilities	Questionnaire	(i) Administrative policies (ii) Supervision (iii) Salary (iv) Interpersonal relationships (v) Working conditions (vi) Achievement (vii) Recognition (viii) The work itself (ix) Responsibility (x) Advancement	Questionnaire based on Herzberg's motivation-hygiene factors 20 items: 2 for each of the 10 Herzberg items	Pilot with 10 subjects (split-half reliability was 0.80)	Not reported	Weak

House [63], *Thesis* (1990)	Cross-sectional survey (single group)	*Country*: USA *Sample size*: *n = *148 *Subjects*: CNAs from 10 NHs	Questionnaire	(i) Motivation factors: achievement, recognition, work itself, responsibility, possibility of growth, and advancement (ii) Hygiene factors: salary, technical supervision, company policy, interpersonal relationships with peers, interpersonal relationships with supervisors, working conditions, security, status, personal life, and interpersonal relationship with nurse	Modified version of the JS instrument developed by Kroen which incorporates motivation/hygiene theory	JS scale has a reliability of 0.84 and the JDS scale has a reliability of 0.79 (as tested by Kroen)	Reported valid in previous studies	Low moderate

Kostiwa [64], *Clinical Gerontologist* (2009)	Cross-sectional survey (single group)	*Country*: USA *Sample size*: *n = *60 *Subjects*: CNAs from 12 residential care facilities	Questionnaire	(i) Service quality (ii) Psychological empowerment	The Benjamin Rose Job Satisfaction Survey (JSS; 18 items)	*α* = 0.93 (overall score)	Not reported	Low moderate

Kovach [9], *Research in Gerontological Nursing* (2010)	Cross-sectional survey (single group)	*Country*: USA *Sample size*: *n = *177 *Subjects*: CNAs in 3 residential care facilities	Questionnaire	(i) Personality traits, for example, adjustment, prudence, likeability, being excitable, being dutiful (ii) Job performance	The General Job Satisfaction Scale (5 items)	The internal consistency of the GJS for this sample was 0.57	Prior evidence of construct validity: negative relations to organizational size and positive relations with job level, tenure, performance, and motivational fit with work	Strong

Kuo [10], *Journal of Clinical Nursing* (2008)	Cross-sectional survey (single group)	*Country*: Taiwan *Sample size*: *n = *114 *Subjects*: NAs from 28 residential care facilities	Questionnaire	(i) Organizational empowerment (ii) Demographic variables, for example, nationality, age, marital status, educational level, work duration at a facility	Short form of the Minnesota Satisfaction Questionnaire (MSQ; 20 items)	*α* = 0.87 (overall score)	Not reported	Low moderate

Lerner [65], *Journal of * *Nursing Administration* (2011)	Cross-sectional survey (single group)	*Country*: USA. *Sample size*: *n = *556 *Subjects*: NAs from 12 skilled nursing facilities	Survey pre- and postintervention	(i) Skilled nursing facility site (ii) Age (iii) Gender (iv) Education (v) Years of experience (vi) Self-esteem (vii) Self-efficacy (viii) Outcome expectations for performance of restorative care activities (ix) Observed performance of restorative activities	Job attitude scale (17 items) measuring 5 components; pay factors, organizational factors, task requirements, job status, and autonomy Response options range from 1 (strongly disagree) to 5 (strongly agree)	Not reported	Validity in previous studies by significant relation between its scores and scores of the Minnesota Satisfaction Scale	Low moderate

Liu [66], *Geriatric Nursing* (2007)	Cross-sectional survey (single group)	*Country*: Taiwan *Sample size*: *n = *244 *Subject*: CNAs from 17 residential care facilities	Questionnaire	(i) Marital status (ii) Full time versus part time (iii) Length of tenure (iv) Feelings toward the job (v) Intention to quit (vi) JS Facet 2 (work performance and rewards)	Designed by author according to relevant theoretical literatures and addressed 5 main dimensions of job satisfaction	*α* = 0.81	Not reported	High moderate

McGilton [67], *Journal of * *Nursing Administration* (2007)	Cross-sectional survey (single group)	*Country*: Canada *Sample size*: *n = *222 *Subjects*: CNAs in 10 LTC facilities	Questionnaire	(i) CNA characteristics (age, gender, education, experience working in LTC, ethnicity [origin of birthplace, Canadian versus non-Canadian and first language, English versus non-English]) (ii) Job stress (iii) Supervisory support	Nursing Job Satisfaction Scale (42 items)	*α* = 0.89 (total scale) *α* = 0.88–0.95 (subscales)	Not reported	High moderate

Parmelee [68], *Journal of American * *Medical Directors Association* (2009)	Cross-sectional survey (single group)	*Country*: USA *Sample size*: *n = *188 *Subjects*: NAs registered at the 2006 conference of the National Association of Health Care Assistants	Questionnaire	(i) Perceived barriers to job performance (ii) Teamwork (iii) Job stress (iv) Respect (v) Workload (vi) Exclusion (vii) New NAs	Benjamin Rose Institute Nurse Assistant Job Satisfaction Scale (18 items)	*α* = 0.95	Not reported	High moderate

Parsons [33], *Journal of Gerontological Nursing* (2003)	Cross-sectional survey (single group)	*Country*: USA *Sample size*: *n = *550 *Subjects*: HCAs from 70 LTC facilities	Questionnaire	(i) Demographics: age, race, sex, marital status, education and education goals, family responsibilities, work characteristics (does not specify what) (ii) Seven factors: (1) task rewards, (2) social rewards, (3) supervision, (4) benefits, (5) personal opportunity, (6) coworker support, (7) salary and management keeping employees informed (iii) Turnover	Developed their own: overall satisfaction (3 items)	Not reported	Not reported	Weak

Proenca [69], *Academy of Management Annual Meeting Proceedings* (2008)	Cross-sectional survey (single group)	*Country*: USA *Sample size*: *n = *129 *Subjects*: CNAs from 6 residential care facilities	Questionnaire	(i) Work-family conflict, burnout (ii) Burnout (iii) Supportive supervision (iv) Supportive coworkers	Subscales from the Job Diagnostic Survey and the Michigan Org. Assessment Questionnaire were used to measure job satisfaction and turnover intentions	*α*'s >0.80	Not reported	Low moderate

Purk [70], *Journal of * *Housing for the Elderly* (2006)	Cross-sectional survey (single group)	*Country*: USA *Sample size*: *n = *34 *Subjects*: CNAs from 5 facilities	Questionnaire	(i) Pay, promotion, supervision, work on present job, people at work (ii) Perceived emotional and physical stress (iii) Intent to quit within the next 3 months (iv) Intent to quit within the next year	The Job Descriptive Index (JDI) and the Job in General Scale (JIG)	Not reported	Not reported	Weak

Ramirez [71], *Journal of Mental Health and Aging* (1998)	Cross-sectional survey (single group)	*Country*: USA *Sample size*: *n = *337 *Subjects*: HCAs from 20 residential care facilities	Structured (face-to-face interviews)	*Work related demands and stressors* (i) NA workload (ii) NA perceived bias (iii) Work environment evaluation *Work resources* (i) NA training (ii) Work-related support *Individual resources* Years working as a NA *SCU Assignment *	Adaption of Cantor and Chichin Job Satisfaction Scale (5 items)	Internal consistency coefficient for the 5-item set was 0.41 in this study	Not reported	Low moderate

Resnick [72], *Geriatric Nursing * (2004)	Quasi-experimental^*∗∗*^ (single-group repeated measure design)	*Country*: USA *Sample size*: *n = *13 *Subjects*: HCAs from 1 facility	Questionnaire	Implementation of the Res-Care pilot intervention (restorative care philosophy)	Job Attitude Scale (17 items)	Not reported	Referred to a previous study, items on the JAS related to items on Minnesota Satisfaction Scale	Low moderate

Simpson [73], *Thesis* (2010)	Cross-sectional survey (single group) (second analysis of Resnick 2007)	*Country*: USA *Sample size*: *n = *504 *Subjects*: CNAs employed in 12 residential care facilities	NA	Individual factors: age, experience Psychosocial factors: self-esteem, self-efficacy, outcome expectations, knowledge of restorative care CNA job performance (i.e., performance of restorative care)	*The Nursing Assistant Job Attitude Scale (NAJAS) * (17 items): 5 components: pay factors, organizational factors, task requirements, job status, and autonomy	*α* = 0.94	Convergence validity: “prior use of the NAJAS in a sample of 286 certified nurse aides resulted in findings similar to those found by other measures of job satisfaction”	High moderate

Snow [74], *Nursing Homes/Long* *Term Care * *Management* (2007)	Cross-sectional survey (single group)	*Country*: USA *Sample size*: *n = *121 *Subjects*: HCAs at assisted living and skilled nursing facilities	Questionnaire	(i) Pursuing education (ii) Expansion of scope of practice	Not reported	Not reported	Not reported	Weak

Solomon [75], *Thesis* (2009)	Cross-sectional survey (single group)	*Country*: USA *Sample size*: *n = *66 *Subjects*: CNAs, 5 RNs, and 1 administrator in one residential care facility	Questionnaire	Leadership characteristics of administrators and registered nurses: (i) Modeling the way (ii) Inspiring a shared vision (iii) Challenging the process (iv) Enabling others to act (v) Encouraging the heart	The Benjamin Rose Nurse Assistant Job Satisfaction Survey	Not reported	Not reported	Low moderate

Tannazzo [76], *Alzheimer's Care Today* (2008)	pre/post-test intervention^*∗∗*^	*Country*: USA *Sample size*: *n = *301 *Subjects*: CNAs from 4 residential care facilities	Questionnaire	Education intervention, knowledge of Alzheimer's	General Job Satisfaction (GJS) (5 items) and a Grau Satisfaction Scale (GSS; 2 items) measuring intrinsic satisfaction and satisfaction with benefits	GJS: *α* = 0.45–0.58 GSS: *α* = 0.81–0.84	Not reported	Low moderate

Thompson [77], *Journal of Gerontological Nursing* (2011)	Cross-sectional survey	*Country*: USA *Sample size*: *n = *40 *Subjects*: NAs in 1 skilled nursing facility	Mailed questionnaire	Work content, quality of care, training, coworkers, supervisors, work demands, workload, rewards, global rating	Adapted Nursing Home Nurse Aide Job Satisfaction Questionnaire [31]	Not reported.	Content validity—instrument based on the literature; a panel of experts and cognitive testing were also conducted	Weak

Tyler [78]^*∗*^, *Health Care Management Review* (2006)	Mixed methods (qualitative ground theory and quantitative cross-sectional survey)	*Country*: USA *Sample size*: *n = *1146 (surveys) *n = *144 (interviews) *n = *37 (participant observations) *Subjects*: CNAs, RNs, management at 20 facilities	Qualitative: ethnographic interviews and participant observations Quantitative: questionnaire	(i) Skill variety (ii) Task identity (iii) Task significance (iv) Autonomy (v) Intrinsic feedback	Modified version of the Job Diagnostic Survey (JDS)	*α* = 0.35–0.71	Not reported	Low moderate/strong

Walborn [79], *Thesis* (1996)	Cross-sectional survey (single group)	*Country*: USA *Sample size*: *n = *185 *Subjects*: HCAs and charge nurses from one residential care facility	Questionnaire	(i) Demographic variables (age, education, number of years since training, years of experience) (ii) Job performance variables (iii) Absenteeism variables (iv) Variables of perceptions of the work environment	The Job Descriptive Index (JDI) and the Job in General Scale (JGS) and 2 items from the Quality of Employment survey (QES) measuring overall JS	JDI: *α* = 0.74 (overall) *α* = 0.67–0.92 (for 5 subscales) JGS: *α* = 0.86 (overall)	Reported as valid in previous studies	Weak

Webb [80], *Thesis* (2003)	Quasi-experimental (nonequivalent control group design with pre- and posttest)^*∗∗*^	*Country*: USA *Sample size*: *n = *178 *Subjects*: CNAs from 2 residential care facilities	Questionnaire	Recognition and rewards training program	The Nurse Assistant Assessment Survey Instrument: Job Satisfaction which was developed by Iowa CareGivers Association and Hill Simonton Bell (1998) (48 items)	*α* = 0.87 (pretest) *α* = 0.88 (posttest)	Content validity by 3 experts	Weak

Yeatts [55]^*∗*^, *The Gerontologist * (2007)	Mixed methods (before-and-after^*∗∗*^ with small amount of qualitative data)	*Country*: USA *Sample size*: ** **not reported *Subjects*: work teams of 5 residential care facilities with intervention implemented and 5 work teams from 5 other residential care facilities as control	Quantitative: questionnaires Qualitative: (i) Participating observations, of over 270 CNA team meetings (ii) Examination of weekly team-meeting summaries for management and management's responses	Empowerment	Index in CNA survey (details of items not reported)	CNA survey indices ranged from 0.60 to 0.85 (specific index for JS not reported)	Factor analysis to determine items in all survey indices	Weak

*Qualitative studies (n = 7) *								

Ball [81], *Journal of Aging Studies* (2009)	Long qualitative ground theory	*Country*: USA *Sample size*: *n = *43 *Subjects*: management staff members and DCWs in 2 ALFs	(i) Participant observation (ii) In-depth and informal interviews	(i) No predefined individual variables (ii) Open-ended interviews were used to find out what individual variables are important from the participants' perspectives	Participant observations and qualitative interviews	N/A	N/A	Strong

Bye [82], *Nursing Homes and Senior* *Citizen Care* (1987)	Qualitative cross-sectional interview	*Country*: USA *Sample size*: *n = *30 *Subjects*: NAs from 3 residential care facilities	Semistructured cross-sectional interview study	(i) No predefined individual variables (ii) Open-ended interviews were used to find out what individual variables are important from the participants' perspectives	Asked participants for their subjective perceptions of what satisfied them in their jobs	N/A	N/A	Weak

Karner [83], *Journal of Gerontological Nursing* (1998)	Qualitative cross-sectional ground theory	*Country*: USA *Sample size*: 17 *Subjects*:CNAs (article focused on CNAs but respondents included other staff members)	Semistructured guided intensive interviews	(i) No predefined individual variables (ii) Open-ended interviews were used to find out what individual variables are important from the participants' perspectives	Asked participants for their subjective perceptions of what impacts their satisfaction	N/A	N/A	Low moderate

Moyle [84], *Journal of Clinical Nursing* (2003)	Qualitative cross-sectional interview study	*Country*: Australia *Sample size*: *n = *13 *Subjects*: CNAs (plus 9 RNs and 5 ENs)	Focus group interviews	(i) No predefined individual variables (ii) Open-ended focus groups were used to find out what individual variables are important from the participants' perspectives	Focus groups: subjective views and opinions of the interviewed individuals or group meanings, respectively	N/A	N/A	Strong

Quinn [85], *Thesis* (2002)	Mixed methods: qualitative long interview study with survey	*Country*: USA *Sample size*: *n = *14 *Subjects*: CNAs of one residential care facility	Semistructured, open-ended interviews	(i) No predefined individual variables (ii) Open-ended interviews were used to find out what individual variables are important from the participants' perspectives	Asked participants for their subjective perceptions of what satisfied them in their jobs. Started with 2 open-ended job satisfaction questions	N/A	N/A	Strong

Tyler [78]^*∗*^, *Health Care Management Review* (2006)	Mixed methods: grounded theory and cross-sectional survey	*Country*: USA *Sample size*: *n = *1146 (surveys) *n = *144 (interviews) *n = *37 (participant observations) *Subjects*: CNAs, RNs, management at 20 facilities	Qualitative: ethnographic interviews and participant observations Quantitative: questionnaire	(i) Skill variety (ii) Task identity (iii) Task significance (iv) Autonomy (v) Intrinsic feedback	Modified version of the Job Diagnostic Survey (JDS)	*α* = 0.35–0.71	Not reported	Low moderate/strong

Yeatts [55]^*∗*^, *The Gerontologist * (2007)	Mixed methods: before-and-after with small amount of qualitative data	*Country*: USA *Sample size*: ** **not reported *Subjects*: CNAs	Quantitative: questionnaires Qualitative: (i) Participating observations, of over 270 CNA team meetings (ii) Examination of weekly team-meeting summaries for management and management's responses	Empowerment	Index in CNA survey (details of items not reported)	CNA survey indices ranged from 0.60 to 0.85 (specific index for JS not reported)	Factor analysis to determine items in all survey indices	Weak

^*∗*^These studies are listed as both quantitative and qualitative as they employed a mixed methods study design.

^*∗∗*^The overall study design is quasi-experimental. The explanatory variables from these studies used in our analysis are the independent variables, not the experimental variable(s).

ALF: assisted living facility, CNA: certified nursing assistant, DCW: direct care worker, EN: enrolled nurses, HCA: health care aides, HPPD: hours per patient day, JS: job satisfaction, LTC: long-term care, NA: nursing assistant, PACE: Program of All-Inclusive Care for the Elderly, RN: registered nurse, and SCU: special care unit.

**Table 3:** Individual factors (reported four or more times).

Category	First author	Significance (S = *p* < .05)	Direction (magnitude)	Methodological quality	Sample size
(1)* Sociodemographics (n = 13 studies) *					

Age (*n* = 12 studies)	Allensworth-Davies [47]	NS		Weak	135
	Blackmon [49]	NS		Weak	188
	Choi [53]	NS		High moderate	2,254
	Friedman [56]	S	+ (*β* = 0.15)	High moderate	349
	Gittell [58]	NS		Low moderate	252
	Grieshaber [60]	NS		Weak	79
	Kuo [10]	NS		Low moderate	114
	Lerner [65]	NS		Low moderate	556
	McGilton [67]	NS		High moderate	222
	Parsons [33]	NS		Weak	550
	Simpson [73]	S	+ (*β* = 0.14)	High moderate	504
	Walborn [79]	S	+ (*r* = 0.218)	Weak	185

Ethnicity (*n* = 7 studies)	Allensworth-Davies [47]	NS		Weak	135
	Blackmon [49]	NS		Weak	188
	Choi [53]	NS		High moderate	2,254
	Kuo [10]	S	+ (*β* = 0.32)	Low moderate	114
	McGilton [67]	S	− (*β* = −0.28)	High moderate	222
	Parsons [33]	NS		Weak	550
	Ramirez [71]	S (for 2/3 races)	− (*β* = −0.14 to −0.20)	Low moderate	337

Gender (*n = *6 studies)	Blackmon [49]	NS		Weak	188
	Gittell [58]	NS		Low moderate	252
	Kuo [10]	NS		Low moderate	114
	Lerner [65]	NS		Low moderate	556
	McGilton [67]	NS		High moderate	222
	Parsons [33]	NS		Weak	550

(2) *Education (n = 17 studies) *					

Level of education/years Education (*n = *10 studies)	Blackmon [49]	NS		Weak	188
	Choi [53]	NS		High moderate	2,254
	Friedman [56]	NS		High moderate	349
	Gittell [58]	NS		Low moderate	252
	Goldwasser [59]	S	−^*∗*^	Weak	27
	Grieshaber [60]	NS (urban)		Weak	79
	Grieshaber [60]	S (suburban)	− (*r* = −0.51)	Weak	79
	Kuo [10]	NS		Low moderate	114
	Lerner [65]	NS		Low moderate	556
	Parsons [33]	NS		Weak	550
	Walborn [79]	S	− (*r* = −0.274)	Weak	185

Special training (*n = *8 studies)	Blackmon [49]	NS		Weak	188
	Braun [51]	S	+^*∗*^	Weak	105
	Ramirez [71]	S	− (*r* = −0.13)	Low moderate	337
	Resnick [72]	NS		Low moderate	13
	Simpson [73]	NS		High moderate	504
	Tannazzo [76]	NS		Low moderate	301
	Thompson [77]	NS		Weak	40
	Webb [80]	NS		Weak	178

(3) *Healthcare provider characteristics (n = 18 studies) *					

Empowerment (*n = *5 studies)	Cready [54] (autonomy in decision making and perceived meaningful work with a feeling of competence to do it)	S	+^*∗*^	Weak	434
	Gruss [61] (perceived control and access to power within the organization)	S	+ (*r* = 0.46)	Low moderate	42
	Kostiwa [64] (transfer of power to nonmanagement employees)	S	+ (*β* = 0.294)	Low moderate	60
	Kuo [10] (perceived support, access to information and resources, opportunity to learn and grow, good relationships with staff)	S	+ (*r* = 0.366)	Low moderate	114
	Yeatts [55] (autonomy in decision making and perceived meaningful work with competence to do it)	NS		Weak	Not reported

Years of experience (*n = *5 studies)	McGilton [67]	NS		High moderate	222
	Lerner [65]	S	+ (*β* = 0.230)	Low moderate	114
	Ramirez [71]	NS		Low moderate	337
	Simpson [73]	NS		High moderate	504
	Walborn [79]	S	+ (*r* = 0.204)	Weak	185

Current position tenure (*n = *3 studies)	Gittell [58]	NS		Low moderate	252
	Grieshaber [60]	NS (urban)		Weak	79
	Grieshaber [60]	S (suburban)	+ (*r* = 0.38)	Weak	79
	Liu [66]	S	− (*β* = −0.14)	High moderate	244

Employment status (rotating, part time, full time) (*n = *4 studies)	Albanese [46]	NS		Weak	255
	Burgio [52]	S	*F*(1,173) = 6.38	Low moderate	178
	Liu [66]	S	− (*β* = −0.15)	High moderate	244
	McGilton [67]	NS		High moderate	222

Autonomy (*n = *3 studies)	Allensworth-Davies [47] (definition not reported)	S	+ (*β* = 0.23)	Weak	135
	Friedman [56] (opportunity to use their own judgment)	NS		High moderate	349
	Friedman [56] (opportunity to organize workload)	S	+ (*β* = 0.17)	High moderate	349
	Tyler [78] (degree to which a job provides independence and discretion in scheduling work and determining ways to carry it out)	S	*∗*	Low moderate/strong	1146

(4) *Personal life (n = 4 studies) *					

Stress (*n* = 4 studies)	Albanese [46]	S	− (*r* = −0.37)	Weak	255
	McGilton [67]	S	− (*β* = −0.19)	High moderate	222
	Parmelee [68]	NS		High moderate	188
	Purk [70]	NS		Weak	34

*∗*: test statistic value not reported; *r*: estimate of the Pearson product-moment correlation coefficient; *β*: in multiple regression, a standardized coefficient indicating the relative weight of a predictor variable.

**Table 4:** Organizational factors (reported four or more times).

Category	First author	Significance (S = *p* < .05)	Direction (magnitude)	Methodological quality	Sample size
(1) *Facility (n = 3 studies) *					

Resources (*n* = 3 studies)	Garland [57]	S	+ (*r* = 0.43)	Low moderate	138
	Kuo [10], information	NS		Low moderate	114
	Kuo [10], resources	S	+ (*β* = 0.32)	Low moderate	114
	Ramirez [71]	S	+ (*β* = 0.24)	Low moderate	337

(2) *Work environment (n = 13 studies) *					

Satisfaction with salary/benefit (*n* = 4 studies)	Choi [53], salary	NS		High moderate	2,254
	Choi [53], benefits	S	OR = 1.14^*∗∗*^	High moderate	2,254
	House [63]	NS		Low moderate	148
	Parsons [33], salary	NS		Weak	550
	Parsons [33], benefits	NS		Weak	550
	Purk [70]	S	*∗*	Weak	34

Job performance (*n* = 4 studies)	Kovach [9]	NS		Strong	177
	Liu [66]	S	+ (*β* = 0.40)	High moderate	244
	Simpson [73]	NS		High moderate	504
	Walborn [79]	NS		Weak	185

Support from coworkers (*n* = 6 studies)	Friedman [56]	NS		High moderate	349
	Kuo [10]	NS		Low moderate	114
	Parmelee [68]	S	− (*β* = −0.145)	High moderate	188
	Parsons [33]	S	+ (*β* = 0.138)	Weak	550
	Proenca [69]	NS		Low moderate	129
	Thompson [77]	S	*∗*	Weak	40

(3) *Workload (n = 5 studies) *					

Workload (*n* = 5 studies)	Berg [48] (perceived strain)	S	− (*r* = −0.38)	Weak	233
	Garland [57]	S	+ (*r* = 0.3)	Low moderate	138
	Parmelee [68]	S	− (*β* = −0.283)	High moderate	188
	Ramirez [71]	S	− (*β* = −0.21)	Low moderate	337
	Thompson [77]	S	*∗*	Weak	40

*∗*: test statistic value not reported; *∗∗*: *χ* ^2^ not reported; *r*: estimate of the Pearson product-moment correlation coefficient; *β*: in multiple regression, a standardized coefficient indicating the relative weight of a predictor variable.

**Table 5:** Individual factor conclusions.

Sociodemographic		

Age	3/12 (25%) reports significant	No relationship with job satisfaction
Ethnicity	3/7 (43%) reports significant	No relationship with job satisfaction
Gender	0/6 (0%) reports significant	No relationship with job satisfaction

Education		

Level of education/years Education	3/11 (27%) reports significant	No relationship with job satisfaction
Special training	2/8 (25%) reports significant	No relationship with job satisfaction

Professional characteristics		

Empowerment	4/5 (80%) reports significant	Positive relationship with job satisfaction
Years of experience	2/5 (40%) reports significant	No relationship with job satisfaction
Current position	2/4 (50%) reports significant	Equivocal relationship with job satisfaction
Employment status	2/4 (50%) reports significant	Equivocal relationship with job satisfaction
Autonomy	3/4 (75%) reports significant	Positive relationship with job satisfaction

Personal life		

Stress	2/4 (50%) reports significant	Equivocal relationship with job satisfaction

**Table 6:** Organizational factor conclusions.

Facility		

Resources	3/4 (75%) reports significant	Positive relationship with job satisfaction

Work environment		

Satisfaction with salary/benefits	2/6 (33%) reports significant	No relationship with job satisfaction
Job performance	1/4 (25%) reports significant	No relationship with job satisfaction
Support from coworkers	3/6 (50%) reports significant	Equivocal relationship with job satisfaction

Workload		

Workload	5/5 (100%) reports significant	Positive relationship with job satisfaction

**Table 7:** Summary of qualitative findings.

Factor	First author	Details
*Individual factors *		

*Education *		
Pursuing education	Snow [74]	CNAs reported they would have greater job satisfaction with more education/expanded skills
Pursuing nursing career	Snow [74]	(i) CNAs pursuing a nursing career reported the highest level of job satisfaction, followed by CNAs with no plans for further education (ii) CNAs pursuing education outside of health care reported the lowest levels of job satisfaction
*Other *		
Feeling needed/useful	Bye [82]	93% stated feeling needed/useful was the most satisfying aspect of their work

*Organizational factors *		

*Facility: resources *		
Equipment and supplies	Quinn [85]	Mainly positive responses, more resources linking to higher job satisfaction
*Facility: other *		
Workplace flexibility	Moyle [84]	Related to job satisfaction
Working on skilled units	Bye [82]	Some enjoyed challenge of working on skilled units
Facility	Bye [82]	Some were happy in their current facility and would not like to go to another facility
Pay satisfaction	Quinn [85]	Typical responses positive in relation to job satisfaction
Benefits satisfaction	Quinn [85]	Many variant responses positive/negative re job satisfaction
Facility's response to needs and concerns	Quinn [85]	Many variant responses positive/negative re job satisfaction
People in management	Quinn [85]	Many variant responses positive/negative re job satisfaction
Admin support	Karner [83]	Contributing to increased job satisfaction—appropriate and kind administrative support; respectful of aides' knowledge
*Work environment *		
Working with unskilled or inappropriately trained staff	Moyle [84]	Related to job dissatisfaction
Working conditions	Holtz [62]	68% of aides said that they were extremely or very important to their job satisfaction
Organizational structure	Karner [83]	Contributing to increased job satisfaction—fair and consistent organizational structures; hands-on training and adequate staff
Recognition/respect	Holtz [62]	77% of aides said that it was extremely or very important
	Quinn [85]	Many variant responses—some say recognition for work is important to job satisfaction and others lead to job dissatisfaction
	Quinn [85]	Typical response negative for quantity of recognition leading to job satisfaction
	Walborn [79]	Nurse aides would like more respect, for example, from family members
Residents	Bye [82]	Most identified their interaction with residents as the most satisfying aspect of their job
	Quinn [85]	Many variant responses, typical response positive in relation to job satisfaction
	Moyle [84]	(i) Related to job satisfaction (ii) Contact with residents promotes enjoyment and job satisfaction (iii) Job satisfaction comes from resident: interactions and appreciation
	Walborn [79]	Interacting with residents was a satisfying aspect of the job
	Karner [83]	Relation with residents was a satisfying aspect of the job
Family member participation in resident care	Karner [83]	Contributing to increased job satisfaction
Interpersonal relationships	Quinn [85]	Typical response positive in relation to job satisfaction
	Holtz [62]	100% of aides said that interpersonal relationships were important or extremely important
	Bye [82]	53% said these were 2nd and 3rd greatest satisfiers
Support from coworkers	Moyle [84]	(i) Good teamwork increases job satisfaction (ii) Job dissatisfaction occurs when staff members are intolerant/upset
	Karner [83]	Contributing to increased job satisfaction
	Quinn [85]	Typical response positive in relation to job satisfaction
Tensions within role expectations	Moyle [84]	Related to job dissatisfaction
Absenteeism	Quinn [85]	Typical responses negative in relation to job satisfaction
Environment (homelike)	Karner [83]	Contributing to increased job satisfaction
Building design	Quinn [85]	Many variant responses positive in relation to job satisfaction
Positive feedback	Tyler [78]	Positive feedback often comes from residents and this type of feedback is more important than feedback received from supervisors
Communication—valued input	Quinn [85]	Many variant responses negative in relation to job satisfaction
Respect	Walborn [79]	Nurse aides would like more respect, for example, from family members
*Supervision *		
Supervision	Holtz [62]	90% of aides said that it was extremely or very important
	Walborn [79]	Nursing assistants would like to be listened to by charge nurses/managers
*Staffing *		
Number of staff and workloads	Quinn [85]	Mainly positive responses with respect to more staff linking to higher job satisfaction
Staffing levels	Moyle [84]	(i) Job satisfaction decreases when tasks and time constraints prevent the opportunity to relate to residents and increases likelihood of error (ii) Dissatisfied with anything that took them away from resident care
Increasing need to be available for overtime	Moyle [84]	(i) Related to job dissatisfaction (ii) Overtime created both job satisfaction and dissatisfaction
*Other: opportunity for learning and advancement *		
Learning and growing on the job	Bye [82]	17% said this was 2nd and 3rd greatest satisfiers
Expansion of scope of practice	Snow [74]	Overall 92% of the certified nursing assistants believed that expansion of their scope of practice would increase their job satisfaction
Advancement	Holtz [62]	48% of aides said that it was extremely or very important
*Other: nature of the job *		
Work itself	Holtz [62]	84% of aides said that it was extremely or very important
	Quinn [85]	Many variant responses in relation to job satisfaction
	Moyle [84]	(i) Laborious tasks (such as documentation) related to job dissatisfaction (ii) Job dissatisfaction occurs when tensions are not recognized in the workplace: managerial staff not listening to concerns

## List of Abbreviations

ARD: Age-related dementia
LTC: Long-term care
RN: Registered nurse
LPN: Licensed practical nurse
RPN: Registered practical nurse
NA: Nursing assistant/aide
CNA: Certified nurse aide.
