# Supplementary material for: Job Satisfaction among Care Aides in Residential Long-Term Care: A Systematic Review of Contributing Factors, Both Individual and Organizational
Source: Nurs Res Pract. 2015 Aug 5;2015:157924. doi: 10.1155/2015/157924 (PMC4541006; doi:10.1155/2015/157924)
Supplement: Supplementary file 1 — Additional file 1 contains a list of studies excluded from the review at the stage of full-text screening and the reasons for their exclusion. Additional file 2 contains the details of the methodological quality assessment conducted on all 42 studies included in the review. Additional file 3 provides a summary of findings with respect to the relationship between job satisfaction and individual and organizational factors that were assessed less than 4 times. Factors assessed 4 or more times are presented in the body of the paper. [file 157924.f1.pdf]

Additional File 1: Excluded Articles Based on Full Text (n=122)

NA= Nurse Aide; LTC=Long-Term Care

**Citation**	**Exclusion Reason**
Abraham, S. E., Friedman, B. A., & Thomas, R. K. (2008). The relationship among union membership, facets of satisfaction and intent to leave: Further evidence on the voice face of unions. *Employee Responsibilities and Rights Journal, 20*(1), 1-11.	This article does not focus on health care aides employed in facility-based LTC settings
Abrahamson, K., Suitor, J. J., Pillemer, K. (2009). Conflict between nursing home staff and residents; families Does it increase burnout?. *Journal of Aging Health, 21*(6), 895-912.	This article is not primary research
Acampora, A. (1993). Satisfaction on the job: The "value" factor. *Journal of Long Term Care Administration*, *21*(2), 17.	Unable to retrieve complete record
Alexander, J. A., Lichtenstein, R., Oh, H. J., & Ullman, E. (1998). A causal model of voluntary turnover among nursing personnel in long-term psychiatric settings. *Research in Nursing & Health, 21*(5), 415-427.	The article does not evaluate the relationship between either personal or job characteristics and overall job satisfaction
Anderson, M. A., Aird, T. R., & Haslam, W. B. (1991). How satisfied are nursing home staff? *Geriatric Nursing, 12*(2), 85-87.	Unable to retrieve complete record
Atchison, J. H. (1996). Perceived job satisfaction factors of nursing assistants employed in midwest nursing homes. Indiana State University. , 63 p. (UMI Order AAI9703909.)	The article does not evaluate the relationship between either personal or job characteristics and overall job satisfaction
Ayalon, L. (2008). Subjective socioeconomic status as a predictor of long-term care staff burnout and positive caregiving experiences. *International Psychogeriatrics 20*(3), 521-537.	The article does not evaluate the relationship between either personal or job characteristics and overall job satisfaction
Barra, M. & Guttman, M. (2012). One less call bell to answer: Improving clinical outcomes in an assisted living facility with bedside shift reports and hourly rounds. UPNAAI Nursing Journal, 8(1), 19-26.	This article does not evaluate the relationship between either personal or job characteristics and overall job satisfaction.
Bayer, M., Bresloff, L., & Curley, D. (1986). Enhancement project: A program to improve the quality of residents' lives. *Geriatric Nursing, 7*(4), 192-195.	The article does not evaluate the relationship between either personal or job characteristics and overall job satisfaction
Bergman, R., Eckerling, S., Golander, H., Sharon, R., & Tomer, A. (1984). Staff composition, job perceptions, and work retention of nursing personnel in geriatric institutions. *International Journal of Nursing Studies, 21*(4), 279-293.	The article does not evaluate the relationship between either personal or job characteristics and overall job satisfaction
Berkenstock, G., Waxman, H. M., & Carner, E. A. (1983). Job turnover and job-satisfaction among nursing-home aides. *Gerontologist, 23*, 267-267.	This article is not primary research
Bernier, S. L., & Small, N. R. (1988). Disruptive behaviors. *Journal of Gerontological Nursing*, *14*(2), 8-13.	The article does not evaluate the relationship between either personal or job characteristics and overall job satisfaction
Birk, S. (2010). Survey shows surprising staff job satisfaction. *Caring for the Ages, 11*(1), 1.	The article does not evaluate the relationship between either personal or job characteristics and overall job satisfaction and is not primary research
Bishop, C. E., Squillace, M. R., Meagher, J., Anderson, W. L., Wiener, J. M. (2009). Nursing home work practices and nursing assistants' job satisfaction. *The*	This article is not primary research

Citation	Exclusion Reason
Gerontologist, 49(5), 611-622.	
Bloom, R. L. (1994). The effect of type of psychological training on nursing staff empathy and attitudes towards geriatric residents. *Dissertation Abstracts International,* 55(06), 2146. (UMI No. 9429104)	This article is not primary research
Boekhorst, S., Willemse, B., Depla, M. F. I. A., Eefsting, J. A., Pot, A. M. (2008). Working in group living homes for older people with dementia: the effects on job satisfaction and burnout and the role of job characteristics. *International Psychogeriatrics, 20*(5), 927-940.	This article does not focus on health care aides employed in facility-based LTC settings
Brannon, D., Cohn, M. D., & Smyer, M. A. (1990). Care giving as work: How nurse's aides rate it. *Journal of Long-Term Care Administration, 18*(1), 10-14.	Unable to retrieve complete record
Brannon, D., Smyer, M. A., Cohn, M. D., Borchardt, L., & Landry, J. A. (1988). Job diagnostic survey of nursing home caregivers: Implications for job redesign. *Gerontologist, 28*(2), 246-252.	The article does not evaluate the relationship between either personal or job characteristics and overall job satisfaction
Brunk, D. (1995). Show some respect. *Contemporary Long Term Care, 18*(4), 32.	This article is not primary research
Buelow, J. R., & Fee, F. A. (2000). Perceptions of care and satisfaction in assisted living facilities. *Health Marketing Quarterly, 17*(3), 13-24.	This article does not focus on health care aides employed in facility-based LTC settings
Casper, S., O'Rourke, N. (2008). The influence of care provider access to structural empowerment on individualized care in long-term-care facilities. *Journal of Gerontology, 63B*(4), S255-S265.	The article does not evaluate the relationship between either personal or job characteristics and overall job satisfaction
Castle, N. G. (2006a). Organizational commitment and turnover of nursing home administrators. *Health Care Management Review, 31*(2), 156-165.	The article does not evaluate the relationship between either personal or job characteristics and overall job satisfaction
Castle, N. G. (2007a). Assessing job satisfaction of nurse aides in nursing homes: The nursing home nurse aide job satisfaction questionnaire. *Journal of Gerontological Nursing, 33*(5), 41-47.	The article does not evaluate the relationship between either personal or job characteristics and overall job satisfaction
Castle, N. G., Degenholtz, H., & Rosen, J. (2006b). Determinants of staff job satisfaction of caregivers in two nursing homes in Pennsylvania. *BMC Health Services Research, 6,* 60.	Unable to separate out NA results from other participant groups
Castle, N. G., Engberg, J., Anderson, R., & Men, A. (2007b). Job satisfaction of nurse aides in nursing homes: Intent to leave and turnover. *Gerontologist, 47*(2), 193-204.	The article does not evaluate the relationship between either personal or job characteristics and overall job satisfaction
Castle, N. G., Bost, F. S. (2009). Perfecting patient care: Integrating principles of process redesign in nursing homes. *Journal of Applied Gerontology, 28*(2), 256-276.	Unable to separate out NA results from other participant groups
Cherry, B., Ashcraft, A., & Owen, D. (2007). Perceptions of job satisfaction and the regulatory environment among nurse aides and charge nurses in long-term care. *Geriatric Nursing, 28*(3), 183-192.	Unable to separate out NA results from other participant groups
Choi, J. (2010). Work-related and personal factors influencing job satisfaction and intent to leave among certified nursing assistants in nursing homes. Dissertation.	This article is the unpublished dissertation version of a study we included
Chou, S., Boldy, D. P., & Lee, A. H. (2002). Measuring	This article does not focus on health care aides

Citation	Exclusion Reason
job satisfaction in residential aged care. *International Journal for Quality in Health Care, 14*(1), 49-54.	employed in facility-based LTC settings
Cohen-Mansfield, J. (1989). Sources of satisfaction and stress in nursing home caregivers: Preliminary results. *Journal of Advanced Nursing, 14*(5), 383-388.	Unable to separate out NA results from other participant groups
Coogle, C. L., Head, C. A., & Parham, I. A. (2006). The long-term care workforce crisis: Dementia-care training influences on job satisfaction and career commitment. *Educational Gerontology, 32*(8), 611-631.	Unable to separate out NA results from other participant groups
Coogle, C. L., Parham, I. A., & Young, K. A. (2007). Job satisfaction and career commitment among nursing assistants providing Alzheimer's care. *American Journal of Alzheimer's Disease and Other Dementias, 22*(4), 251-260.	Unable to separate out NA results from other participant groups
Coogle, C. L., Parham, I. A., & Rachel, C. A. (2011). Job Satisfaction and Career Commitment Among Alzheimer's Care Providers: Addressing Turnover and Improving Staff Empowerment. *American Journal of Alzheimer's Disease and Other Dementias, 26*(7), 521-527.	The analysis/results were not sorted by setting and included assisted living facilities, nursing homes, adult day care centers, home care agencies and hospitals.
Cortese, C. G. (2007). Job satisfaction of Italian nurses: An exploratory study. *Journal of Nursing Management, 15*(3), 303-312.	This article does not focus on health care aides employed in facility-based LTC settings
Culp, K., Ramey, S., Karlman, S. (2008). Iowa certified nursing assistants study: Self-reported ratings of the nursing home work environment. *Research in Gerontological Nursing, 1*(2), 87-96.	The article does not evaluate the relationship between either personal or job characteristics and overall job satisfaction
Day, L. (1989). Automated staff scheduling in long-term care facilities. *Nursing Management, 20*(3), 76.	This article does not focus on health care aides employed in facility-based LTC settings.<br>The article does not evaluate the relationship between either personal or job characteristics and overall job satisfaction.<br>The article is not primary research
Decker, F. H., Harris-Kojetin, L. D., Bercovitz, A. (2009). Intrinsic job satisfaction, overall satisfaction, and intention to leave the job among nursing assistants in nursing homes. *The Gerontologist, 49*(5), 596-610.	The article is not primary research
de Jonge, J., van Breukelen,Gerard J. P., Landeweerd, J. A., & Nijhuis, F. J. N. (1999). Comparing group and individual level assessments of job characteristics in testing the job demand-control model: A multilevel approach. *Human Relations, 52*(1), 95.	Unable to separate out NA results from other participant groups
de Jonge, J. & Schaufeli, W. R. (1998). Job characteristics and employee well-being: A test of Warr's Vitamin Model in health care workers using structural equation modelling. *Journal of Organizational Behavior, 19*, 387-407.	Unable to separate out NA results from other participant groups
DeMouy, B. (1999). Voice of experience. *Contemporary Long Term Care, 22*(5), 46-48.	The article does not evaluate the relationship between either personal or job characteristics and overall job satisfaction.<br>The article is not primary research
de Veer, A. J., Francke, A. L., Struijs, A., & Willems, D. L. (2012). Determinants of moral distress in daily nursing practice: A cross sectional correlational	Job satisfaction is not the dependent variable. Moral distress is the dependent variable and the sample is mixed without separate analysis

Citation	Exclusion Reason
questionnaire survey. *International Journal of Nursing Studies*.	
Dill, J., Morgan, J., Kelly, C. (2008). The revolving door: Supervision, job satisfaction, and retention among nursing assistants. *The Gerontologist, 48*(III), 127.	Unable to separate out NA results from other participant groups
Dillard, B. G., & Feather, B. L. (1991). The association between attitudes and job-satisfaction - a study of in-home care aides. *Educational Gerontology, 17*(3), 209-218.	This article does not focus on health care aides employed in facility-based LTC settings
Duggleby, W., Cooper, D., Penz, K. (2009). Hope, self-efficacy, spiritual well-being and job satisfaction. *Journal of Advanced Nursing, 65*(11), 2376-2385.	This article does not focus on health care aides employed in facility-based LTC settings
Edwards, D. J. (2003). Study: Staff satisfaction key to retention. *Nursing Homes, 52*(10), 11.	The article is not primary research
Engstrom, M., Ljunggren, B., Lindqvist, R., & Carlsson, M. (2005). Staff perceptions of job satisfaction and life situation before and 6 and 12 months after increased information technology support in dementia care. *Journal of Telemedicine and Telecare, 11*(6), 304-309.	Unable to separate out NA results from other participant groups
Engström, M., Skytt, B., & Nilsson, A. (2011). Working life and stress symptoms among caregivers in elderly care with formal and no formal competence. *Journal of Nursing Management, 19*(6), 732-741.	Did not examine what predicts job satisfaction. Main purpose was to compare "no formal competence" workers (care aides) to "formal competence workers" (LPNs)
Ejaz, F. K., Noelker, L. S., Menne, H. L., Bagaka's, J. G. (2008). The impact of stress and support on direct care worker's job satisfaction. *The Gerontologist, 48* (I), 60-70.	Unable to separate out NA results from other participant groups
Furnham, A., Petrides, K. V., Jackson, C. J., & Cotter, T. (2002). Do personality factors predict job satisfaction? *Personality and Individual Differences, 33*(8), 1325-1342.	This article does not focus on health care aides employed in facility-based LTC settings
Garland, T. N., Gipson, G. A., Oyabu, N., University of Akron & Andrus Foundation. (1987). Nurse assistant as primary provider of health and social care for older adults: Skills and competencies in long term care.	Unable to retrieve complete record
Glister, S. D., & McCracken, A. L. (1991). Unique teaching opportunity: A specialized facility for persons with Alzheimer's disease. *Educational Gerontology, 17*(6), 621-629.	This article does not focus on health care aides employed in facility-based LTC settings.<br>The article does not evaluate the relationship between either personal or job characteristics and overall job satisfaction.<br>The article is not primary research
Grau, L., Chandler, B., Burton, B., & Kolditz, D. (1991). Institutional loyalty and job satisfaction among nurse aides in nursing homes. *Journal of Aging and Health, 3*(1), 47-65.	The article does not evaluate the relationship between either personal or job characteristics and overall job satisfaction
Haggstrom, E., Skovdahl, K., Flackman, B., Kihlgren, A. L., & Kihlgren, M. (2005). Work satisfaction and dissatisfaction--caregivers' experiences after a two-year intervention in a newly opened nursing home. *Journal of Clinical Nursing, 14*(1), 9-19.	Unable to separate out NA results from other participant groups
Hasson, H., & Arnetz, J. E. (2008). Nursing staff competence, work strain, stress and satisfaction in elderly care: A comparison of home-based care and nursing homes: Older people and long-term care.	Unable to separate out NA results from other participant groups

Citation	Exclusion Reason
Journal of Clinical Nursing, 17(4), 468-481.	
Hays, A. M., & Dowling-Williams, T. (1997). Perceptions of job satisfaction in a long term care facility: A comparison between a dedicated Alzheimer's unit and non-Alzheimer's units. *American Journal of Alzheimer's Disease, 12*(1), 35-39.	Unable to separate out NA results from other participant groups
Helmer, F. T., Olson, S. F., & Heim, R. I. (1993). Strategies for nurse aide job satisfaction. *Journal of Long Term Care Administration, 21*(2), 10-14.	The article does not evaluate the relationship between either personal or job characteristics and overall job satisfaction
Holland, B. E., Pariser, D., Gillette, P., & Wiegand, M. (2007). Relationship between perceived care-giving burden and job satisfaction in nursing assistants caring for memory impaired residents in long term care. *Kentucky Nurse, 55*(1), 10-10.	This article is not primary research
Jervis, L. L. (2001). The pollution of incontinence and the dirty work of caregiving in a U.S. nursing home. *Medical Anthropology Quarterly, 15*(1), 84-99.	The article does not evaluate the relationship between either personal or job characteristics and overall job satisfaction
Kaasalainen, S. (2002). Staff development and long-term care of patients with dementia. *Journal of Gerontological Nursing, 28*(7), 39.	The article does not evaluate the relationship between either personal or job characteristics and overall job satisfaction.<br>This article is not primary research
Kane, R. A., Caplan, A. L., Urv-Wong, E. K., Freeman, I. C., Aroskar, M. A., & Finch, M. (1997). Everyday matters in the lives of nursing home residents: Wish for and perception of choice and control. *Journal of the American Geriatrics Society, 45*(9), 1086-1093.	The article does not evaluate the relationship between either personal or job characteristics and overall job satisfaction
Karsh, B., Booske, B. C., & Sainfort, F. (2005). Job and organizational determinants of nursing home employee commitment, job satisfaction and intent to turnover. *Ergonomics, 48*(10), 1260-1281.	Unable to separate out NA results from other participant groups
Kemper, P., Heier, B., Barry, T., Brannon, D., Angelelli, J., Vasey, J. et al. (2008). What do direct care workers say would improve their jobs? Differences across settings. *The Gerontologist, 48*(I), 17-25.	This article does not focus on health care aides employed in facility-based LTC settings.<br>The article does not evaluate the relationship between either personal or job characteristics and overall job satisfaction
Khatusky, G., Wiener, J. M., Anderson, W. L. (2010). Immigrant and non-immigrant certified nursing assistants in nursing homes: How do they differ? *Journal of Aging & Social Policy, 22*(3), 267-287.	The article does not evaluate the relationship between either personal or job characteristics and overall job satisfaction
Komarek, A. G. (2004). Creating a healing environment. *Nursing Homes: Long Term Care Management, 53*(10), 78-81.	This article does not focus on health care aides employed in facility-based LTC settings.<br>The article does not evaluate the relationship between either personal or job characteristics and overall job satisfaction.<br>The article is not primary research
Kruzich, J. M., Clinton, J. F., & Kelber, S. T. (1992). Personal and environmental influences on nursing home satisfaction. *The Gerontologist, 32*(3), 342-350.	This article does not focus on health care aides employed in facility-based LTC settings.<br>The article does not evaluate the relationship between either personal or job characteristics and overall job satisfaction
Lapane, K. L., & Hughes, C. M. (2007). Considering the employee point of view: Perceptions of job satisfaction	The article does not evaluate the relationship between either personal or job characteristics and overall job

Citation	Exclusion Reason
and stress among nursing staff in nursing homes. *Journal of the American Medical Directors Association, 8*(1), 8-13.	satisfaction
Maas, M., Buckwalter, K. C., Swanson, E., & Mobily, P. R. (1994). Training key to job satisfaction. *Journal of Long-Term Care Administration, 22*(1), 23-26.	This article does not focus on health care aides employed in facility-based LTC settings
Mackenzie CS, Poulin PA, Seidman-Carlson R. (2006) A brief mindfulness-based stress reduction intervention. *Applied Nursing Research, 19*(2):105-9.	Unable to separate out NA results from other participant groups
McGilton, K., Irwin-Robinson, H., Boscart, V., & Spanjevic, L. (2006). Communication enhancement: Nurse and patient satisfaction outcomes in a complex continuing care facility. *Journal of Advanced Nursing, 54*(1), 35-44.	Unable to separate out NA results from other participant groups
Mercer, S. O., Heacock, P., & Beck, C. (1994). Nurse's aides in nursing homes: A study of caregivers. *Journal of Women and Aging, 6* (1/2), 107-121.	Unable to retrieve complete record
Monahan, R. S., & McCarthy, S. (1992). Nursing home employment: The nurse's aide's perspective. *Journal of Gerontological Nursing, 18*(2), 13-16.	The article does not evaluate the relationship between either personal or job characteristics and overall job satisfaction
Moniz-Cook, E., Woods, R., & Gardiner, E. (2000). Staff factors associated with perception of behaviour as "challenging" in residential and nursing homes. *Aging and Mental Health, 4*(1), 48-55.	The article does not evaluate the relationship between either personal or job characteristics and overall job satisfaction
Moreno, D. L. (2002). A measurement of job satisfaction indicators of North Dakota long term health care givers. *Dissertation Abstracts International, 64*(04), 1686. (UMI No. 3088051)	The article does not evaluate the relationship between either personal or job characteristics and overall job satisfaction
Morgan, D. G., Semchuk, K. M., Stewart, N. J., & D'Arcy, C. (2002). Job strain among staff of rural nursing homes: A comparison of nurses, aides, and activity workers. *Journal of Nursing Administration, 32*(3), 152-161.	The article does not evaluate the relationship between either personal or job characteristics and overall job satisfaction
Nancarrow, S. (2007). The impact of intermediate care services on job satisfaction, skills and career development opportunities. *Journal of Clinical Nursing, 16*(7), 1222-1229.	This article does not focus on health care aides employed in facility-based LTC settings
Nier, L. (1996). On-the-job injury tied to job satisfaction. *Provider, 22*(6), 47-48.	The article does not evaluate the relationship between either personal or job characteristics and overall job satisfaction.<br>This article is not primary research
Noelker, L. S., Ejaz, F. K., Menne, H. L., Bagaka's, J. G. (2009). Factors affecting frontline worker's satisfaction with supervision. *Journal of Aging & Health, 21*(1), 85-101.	Unable to separate out NA results from other participant groups
Nwosu, M. C. (2006). Relationship between nurse assistants' job satisfaction and perception of quality. *Dissertation Abstracts International, 67*(10). (UMI No. 3237388)	The article does not evaluate the relationship between either personal or job characteristics and overall job satisfaction
Olson, D. M. How do leadership practices influence nursing facility employee satisfaction? A multi-level structural equation model analysis of long-term care quality leadership practices. *Dissertation Abstracts International, 61*(09), p. 4640. (UMI No. 9989141)	Problem with how job satisfaction was defined/ measured

Citation	Exclusion Reason
Ott, M., & van Dijk, H. (2005). Effects of HRM on client satisfaction in nursing and care for the elderly. *Employee Relations, 27*(4/5), 413.	Unable to separate out NA results from other participant groups
Oyabu, N. (1989). Influences on job satisfaction and self-esteem among nurse assistants employed at nursing homes. University of Akron. , 341 p. (UMI Order PUZ8925042.)	Unable to retrieve complete record
Pablo, R. Y. (1976). Job satisfaction in a chronic care facility. *Dimensions in Health Service, 53*(1), 36-39.	This article does not focus on health care aides employed in facility-based LTC settings
Pearson, A., Hocking, S., Mott, S., & Riggs, A. (1992). Skills mix in Australian nursing homes. *Journal of Advanced Nursing, 17*(7), 767-776.	The article does not evaluate the relationship between either personal or job characteristics and overall job satisfaction
Pennington, K., Scott, J., & Magilvy, K. (2003). The role of certified nursing assistants in nursing homes. *Journal of Nursing Administration*, *33*(11), 578-584.	The article does not evaluate the relationship between either personal or job characteristics and overall job satisfaction
Pfefferle, S. G., Weinberg, D. B. (2008). Certified nurse assistants making meaning of direct care. *Qualitative Health Research*, *18*(7), 952-961.	The article does not evaluate the relationship between either personal or job characteristics and overall job satisfaction
Pillemer, K., Meador, R., Henderson, C., Robison, J., Hegeman, C., Graham, E., Schultz, L. (2008). A facility specialist model for improving retention of nursing home staff: Results from a randomized, controlled study. *The Gerontologist, 48*(I), 80-89.	The article does not evaluate the relationship between either personal or job characteristics and overall job satisfaction
Probst, J. C., Baek, J. D., Laditka, S. B. (2010). The relationship between workplace environment and job satisfaction among nursing assistants: findings from a national survey. *Journal of the American Medical Directors Association, 11*(4), 246-252.	This article is not primary research
Rai, G. S. (2012). Job Satisfaction Among Long Term Care Staff: Bureaucracy Isn't Always Bad. *Administration in Social Work*, (just-accepted).	Sample is "long term care staff members," not solely care aides.
Raikkonen, O., Perala, ML, Kahanpaa, A. (2007). Staffing adequacy, supervisory support and quality of care in long-term care settings: staff perceptions. *Journal of Advanced Nursing, 60*(6), 615-626.	The article does not evaluate the relationship between either personal or job characteristics and overall job satisfaction
Rakovski, C. C., Price-Glynn, K. (2010). Caring labour, intersectionality and worker satisfaction: an analysis of the National Nursing Assistant Study (NNAS). *Sociology of Health & Illness, 32*(3), 400-414.	This article is not primary research
Ramarajan, L., Barsade, S. G., Burack, O. R. (2008). The influence of organizational respect on emotional exhaustion in the human services. *The Journal of Positive Psychology, 3*(1), 4-18.	The article does not evaluate the relationship between either personal or job characteristics and overall job satisfaction
Remsburg, R. E., Armacost, K. A., & Bennett, R. G. (1999). GN management. Improving nursing assistant turnover and stability rates in a long-term care facility. *Geriatric Nursing, 20*(4), 203-208.	Unable to separate out NA results from other participant groups
Resnick, B., Petzer-Aboff, I., Galik, E., Russ, K., Cayo, J., Simpson, M., et al. (2008). Barriers and benefits to implementing a restorative care intervention in nursing homes. *Journal of the American Medical Directors Association, 9*(2), 102-108.	The article does not evaluate the relationship between either personal or job characteristics and overall job satisfaction
Resnick, B., Rogers, V., Galik, E., & Gruber-Baldini, A. L. (2007). Measuring restorative care provided by	The article does not evaluate the relationship between either personal or job characteristics and overall job

Citation	Exclusion Reason
nursing assistants: Reliability and validity of the restorative care behavior checklist. *Nursing Research, 56*(6), 387-398.	satisfaction
Riggs, C. J., & Rantz, M. J. (2001). A model of staff support to improve retention in long-term care. *Nursing Administration Quarterly, 25*(2), 43-54.	This article is not primary research
Robinson, J., & Pillemer, K. (2007). Job satisfaction and intention to quit among nursing home nursing staff: Do special care units make a difference? *Journal of Applied Gerontology, 26*(1), 95-112.	Unable to separate out NA results from other participant groups
Rubin, G., Balaji, R. V., Barcikowski, R. (2009). Barriers to nurse/nursing aide communication: the search for collegiality in a southeast Ohio nursing home. *Journal of Nursing Management, 17*(7), 822-832.	Unable to separate out NA results from other participant groups
Ryan, T., Nolan, M., Enderby, P., & Reid, D. (2004). 'Part of the family': Sources of job satisfaction amongst a group of community-based dementia care workers. *Health and Social Care in the Community, 12*(2), 111-118.	This article does not focus on health care aides employed in facility-based LTC settings
Simons, K., & Jankowski, T. B. (2008). Factors influencing nursing home social workers' intentions to quit employment. *Administration in Social Work, 32*(1), 5.	This article does not focus on health care aides employed in facility-based LTC settings
Sloane, P. D., Williams, C. S., Zimmerman, S. (2010). Immigrant status and intention to leave of nursing assistants in U.S. Nursing Homes. *Journal of the American Geriatrics Society, 58*(4), 731-737.	This article is not primary research
Smit, D., B. M. Willemse, J. de Lange and A. M. Pot (2010). Living arrangements for people with dementia study: nursing staff well-being and quality of care... Fourth European Nursing Congress. *Journal of Clinical Nursing, 19*: 13-13.	This is a conference abstract
Spoor, E., J. de Jonge and J. P. H. J. Hamers (2010). The DIRECT-project: interventions to improve job-related outcomes in nursing homes... Fourth European Nursing Congress. *Journal of Clinical Nursing, 19*: 45-45.	This is a conference abstract
Squillace, M. R., Remsburg, R. E., Harris-Kojetin, L. D., Bercovitz, A., Rosenoff, E., et al. (2009). The national nursing assistant survey: Improving the evidence base for policy initiatives to strengthen the certified nursing assistant workforce. *The Gerontologist, 49*(2), 185-197.	The article does not evaluate the relationship between either personal or job characteristics and overall job satisfaction
Stearns, S. C., D'Arcy, L. P. (2008). Staying the course: Facility and profession retention among nursing assistants in nursing homes. *Journal of Gerontology, 63B*(3), S113-S121.	The article does not evaluate the relationship between either personal or job characteristics and overall job satisfaction.<br>This article is not primary research
Stone, E. F. (1976). The moderating effect of work related values on job scope-job satisfaction relationship. *Organizational Behavior & Human Performance, 15(2),* 147-167.	This article does not focus on health care aides employed in facility-based LTC settings
Tak, S., Sweeney, M. H., Baron, S., Calvert, G. M. (2010). Workplace assaults on nursing assistants in US nursing homes: a multilevel analysis. *American Journal of Public Health, 100*(10), 1938-1945.	This article is not primary research
Tellis-Nayak, V. (2007). A person-centered workplace:	The article does not evaluate the relationship between

Citation	Exclusion Reason
The foundation for person-centered caregiving in long-term care. *Journal of the American Medical Directors Association, 8*(1), 46-54.	either personal or job characteristics and overall job satisfaction
Temple, A., Dobbs, D., Ross, A. (2009). Exploring correlates of turnover among nursing assistants in the National Nursing Home Survey. *Health Care Management Review, 34*(2), 182-190.	The article does not evaluate the relationship between either personal or job characteristics and overall job satisfaction
Tveito, T. H., Eriksen, H. R. (2008). Integrated health programme: a workplace randomized controlled trial. *Journal of Advanced Nursing, 65*(1), 110-119.	The article does not evaluate the relationship between either personal or job characteristics and overall job satisfaction
Ullrich, R. A. (1978). Herzberg revisited: Factors in job dissatisfaction. *Journal of Nursing Administration, 8*(10), 19-24.	This article does not focus on health care aides employed in facility-based LTC settings
Verbeek, H., Zwakhalen, S. M., van Rossum, E., Ambergen, T., Kempen, G. I., Hamers, J. P. (2010). Dementia care redesigned: Effects of small-scale living facilities on residents, their family caregivers, and staff. *Journal of the American Medical Directors Association, 11*(9), 662-670,	Unable to separate out NA results from other participant groups
vonDras, D. D. (2009). Workplace stress and ethical challenges experienced by nursing staff in a nursing home. *Educational Gerontology, 35*(4), 321-339.	Unable to separate out NA results from other participant groups
WaXman, H. M., Carner, E. A., & Berkenstock, G. (1984). Job turnover and job satisfaction among nursing home aides. *Gerontologist, 24*(5), 503-509.	The article does not evaluate the relationship between either personal or job characteristics and overall job satisfaction
Will, K., & Simmons, J. (1999). Ohio CNAs speak out. Enjoyment ranks high, pay low in satisfaction study. *Provider, 25*(10), 107.	The article does not evaluate the relationship between either personal or job characteristics and overall job satisfaction
Wilson, R. W., & Patterson, M. A. (1988). Perceptions of stress among nursing personnel on dementia units. *American Journal of Alzheimer's Care and Related Disorders and Research, 3*(4), 34-39.	The article does not evaluate the relationship between either personal or job characteristics and overall job satisfaction
Yeatts, D. E., Cready, C., Swan, J., Shen, Y. (2010). The perception of “training availability” among certified nurse aides: Relationship to CAN performance, turnover, attitudes, burnout, and empowerment. *Gerontology & Geriatrics Education, 31*(2), 115-132.	The article does not evaluate the relationship between either personal or job characteristics and overall job satisfaction
Yeh, S.C. (2003). Nurse aid job satisfaction in long term care facilities. *Show Chwan Medical Journal, 4* (1), 35-45.	Unable to retrieve complete record
Zawacki, R. A., Shahan, R., & Carey, M. (1995). Who has higher job satisfaction: Male or female nurses? *Nursing Management, 26*(1), 54.	Unable to separate out NA results from other participant groups
Zeller, J.M., Lamb, K. (2011). Mindfulness Meditation to Improve Care Quality and Quality of Life in Long-Term Care Settings. *Geriatric Nursing, 32*(2), 114-118.	The article does not evaluate the relationship between either personal or job characteristics and overall job satisfaction.<br>This article is not primary research
Zimmerman, S., Williams, C. S., Reed, P. S., Boustani, M., Preisser, J. S., Heck, E., et al. (2005). Attitudes, stress, and satisfaction of staff who care for residents with dementia. *The Gerontologist, 45*(Suppl. 1), 96-105.	Unable to separate out NA results from other participant groups
Zonteck, T.L, Isernhagen, J.C., Ogle, B.R., (2009).	The article does not evaluate the relationship between

Citation	Exclusion Reason
Psychosocial factors contributing to occupational injuries among direct care workers. *Journal of American Association of Occupational Health Nurses*, *57*(8), 338-347.	either personal or job characteristics and overall job satisfaction

Additional File 2-1: Quality assessment for studies using a cross-sectional study design

					Overall Quality Assessment Rating			
**First Author (Year)**	**Sampling**	**Measurement**	**Statistical Analysis and Conclusions**	**Overall/Possible Score**	**Weak (≤0.50)**	**Low Moderate (0.51-0.65)**	**High Moderate (0.66-0.79)**	**Strong (≥0.80)**
Albanese[53] (1995)	3/6	1/3	2/4	6/13 (0.46)	X			
Allensworth-Davies[54] (2007)	3/6	0/3	3/4	6/13 (0.46)	X			
Berg[55] (1976)	1/6	1/3	3/4	5/13 (0.38)	X			
Burgio[56] (2004)	3/8	2/3	3/4	8/15 (0.53)		X		
Choi[57] (2012)	6/8	0/3	4/4	10/15 (0.67)			X	
Cready[58] (2008)	3/8	1/3	2/4	6/13 (0.46)	X			
Friedman[59] (1999)	4/8	2/3	4/4	10/15 (0.67)			X	
Garland [60](1989)	4/6	1/3	2/4	7/13 (0.54)		X		
Gittell[61] (2008)	3/6	2/3	2/4	7/13 (0.54)		X		
Grieshaber[62](1995)	2/6	2/3	1/4	5/13 (0.38)	X			
Gruss[63] (2007)	3/6	1/3	3/4	7/13 (0.54)		X		
Holtz[64] (1982)	2/6	1/3	0/1	3/10 (0.30)	X			
House[92] (1990)	4/6	2/3	1/4	7/13 (0.54)		X		
Kostiwa[66] (2009)	4/8	2/3	3/4	9/15 (0.60)		X		
Kovach[9] (2010)	5/6	2/3	4/4	11/13 (0.85)				X
Kuo[10] (2008)	4/6	1/3	3/4	8/13 (0.62)		X		
Liu[67] (2007)	5/6	2/3	2/4	9/13 (0.69)			X	

					Overall Quality Assessment Rating			
**First Author (Year)**	**Sampling**	**Measurement**	**Statistical Analysis and Conclusions**	**Overall/Possible Score**	**Weak (≤0.50)**	**Low Moderate (0.51-0.65)**	**High Moderate (0.66-0.79)**	**Strong (≥0.80)**
McGilton[68] (2007)	4/6	2/3	3/4	9/13 (0.69)			**X**	
Parmalee[69] (2009)	4/8	2/3	4/4	10/15 (0.67)			**X**	
Parsons[33] (2003)	3.5/6	0/3	3/4	6.5/13 (0.50)	**X**			
Proenca[70] (2008)	3/6	1/3	3/4	7/13 (0.54)		**X**		
Purk[71] (2006)	2/6	0/3	2/4	4/13 (0.31)	**X**			
Ramirez[72] (1998)	5.5/6	0/3	3/4	8.5/13 (0.65)		**X**		
Simpson[73] (2010)	5/8	2/3	4/4	11/15 (0.73)			**X**	
Snow[74] (2007)	1/6	0/3	0/1	1/13 (0.08)	**X**			
Solomon[75] (2009)	4/8	1/3	3/4	8/15 (0.53)		**X**		
Thompson[76] (2011)	1/8	1/3	3/4	5/15 (0.33)	**X**			
Tyler[77]* (2006)	4/6	1/3	2/4	7/13 (0.54)		**X**		
Walborn[78] (1996)	2/6	2/3	2/4	6/13 (0.46)	**X**			
**Total (n=29)**					**11**	**11**	**6**	**1**

*Study included in two categories.

Additional File 2-2: Quality assessment results for studies using a before-and-after study design

First Author (Year)	Sampling	Design	Control of Confounders	Data Collection and Outcome Measurement	Statistical Analysis and Conclusions	Drop outs	Overall/ Possible Score	Overall Quality Assessment Rating: Weak (≤0.50)	Low Moderate (0.51-0.65)	High Moderate (0.66-0.79)	Strong (≥0.80)
Blackmon[80] (1993)	1/4	1/2	2/4	0/3	3/4	1/1	8/18 (0.44)	X			
Braun[81] (1997)	0/4	1/2	2/4	0/3	2/4	0/1	5/18 (0.28)	X			
Lerner[82] (2011)	3/4	1/2	2/4	1/3	4/4	0/1	11/18 (0.61)		X		
Resnick[83] (2004)	0/4	2/2	2/4	1/3	3/3	1/1	9/17 (0.53)		X		
Tannazzo[84] (2008)	2/3	2/2	0/4	2/3	3/4	0/1	9/17 (0.53)		X		
Webb[85] (2003)	1/4	1/2	2/4	2/3	2/4	0/1	8/18 (0.44)	X			
Yeatts[86]* (2007)	1/4	1/2	4/4	1/3	2/4	0/1	9/18 (0.50)	X			
**Total (n=7)**								**4**	**3**	**0**	**0**

Note: Possible score is not the same for all studies since all quality assessment questions were not applicable to all studies.

*Study included in two categories.

Additional File 2-3: Quality assessment results for studies using a randomized controlled trial study design

								Overall Quality Assessment Rating			
**First Author (Year)**	**Selection Bias**	**Study Design (Allocation Bias)**	**Confounders**	**Blinding**	**Data Collection Methods**	**Withdrawals and Drop-Outs**	**Over all Score**	**Weak (1-1.5)**	**Low Moderate (1.6-2.0)**	**High Moderate (2.1-2.5)**	**Strong (>2.5)**
Goldwasser [79] (1996)	1	1	1	1	3	2	9/6 (1.5)	**X**			
**Total (n=1)**								**1**	**0**	**0**	**0**

Additional File 2-4: Quality assessment results for studies using a qualitative study design

												Overall Quality Assessment Rating			
**First Author (Year)**	**1**	**2**	**3**	**4**	**5**	**6**	**7**	**8**	**9**	**10**	**Overall/ Possible Score**	**Weak (≤0.50)**	**Low Mod (0.51-0.65)**	**High Mod (0.66-0.79)**	**Strong (≥0.80)**
Ball[87] (2009)	1/1	1/1	1/1	0.5/1	1/1	1/1	0/1	1/1	1/1	1/1	8.5/10 (0.85)				**X**
Bye[88] (1987)	1/1	1/1	1/1	0/1	0.5/1	0/1	0/1	0.5/1	0.5/1	0.5/1	5/10 (0.50)	**X**			
Karner [89] (1998)	1/1	1/1	1/1	0/1	1/1	0/1	0/1	0.5/1	1/1	0.5/1	6/10 (0.60)		**X**		
Moyle [90] (2003)	1/1	1/1	1/1	1/1	1/1	0/1	0.5/1	0.5/1	1/1	1/1	8/10 (0.80)				**X**
Quinn [91] (2002)	1/1	1/1	1/1	1/1	1/1	1/1	0.5/1	1/1	1/1	1/1	9.5/10 (0.95)				**X**
Tyler [77]* (2006)	1/1	1/1	1/1	1/1	1/1	0/1	1/1	1/1	1/1	1/1	9/10 (0.90)				**X**
Yeatts [86]* (2007)	1/1	1/1	0.5/1	1/1	0.5/1	0/1	0/1	0/1	0/1	0/1	4/10 (0.40)	**X**			
**Total (n=7)**												**2**	**1**	**0**	**4**

*Study included in two categories.

1: Clear statement of research aims
2: Qualitative methodology appropriate
3: Research design appropriate to address aims
4: Research design appropriate to address aims
5: Data collected in a way that addressed the research issue
6: Relationship between researcher and participants
7: Ethical issues
8: Data analysis sufficiently rigorous
9: Clear statement of findings

10: How valuable is research
Note: Possible score is not the same for all studies since all quality assessment questions were not applicable to all studies.

Additional File 3-1: Individual Characteristics (reported *less* than 4 times)

Category	First Author	Significance (S = p<.05)	Direction (Magnitude)
**1. SOCIO-DEMOGRAPHICS (n=5 STUDIES)**			
First language<br>(n=2 studies)	Gittell[54]<br><br>McGilton[63]	NS<br><br>S	 <br>+ (β = 0.17)
Marital status<br>(n=3 studies)	Kuo[61]<br><br>Liu[62]<br>Parsons[30]	NS<br><br>S<br>NS	 <br>- (β = -0.29)
**2. EDUCATION (n=4 STUDIES)**			
Wish for more training	Garland[53]	NS	
Qualifications - Feel qualified	Garland[53]	S	+ (r = 0.27)
Qualifications - Recognition/rewards training program	Webb[80]	NS	
Education goals	Parsons[30]	NS	
Perceived need for education	Berg[48]	NS	
**3. PROFESSIONAL CHARACTERISTICS (n=4 STUDIES)**			
Length of employment	Blackmon[75]	NS	
Work duration at a facility	Kuo[61]	NS	
Job perceptions (feelings toward the job)	Liu[62]	S	+ (β = 0.55)
Intention to quit	Liu[62]	S	- (β = -0.21)
Years since training	Walborn[73]	NS	
**4. PERSONAL LIFE (n=12 STUDIES)**			
Job interferes with family life/Family life interferes with job	Garland[53]	S	+ (r = 0.18/ r = 0.17)
Family/work conflict<br>(n=2 studies)	Garland[53]<br>Proenca[65]	S<br>S	+ (r = 0.24)<br>- (β = -0.34)
Number of children living with NA	Albanese[46]	NS	
CNA satisfaction with religious life	Albanese[46]	S	+ (r = 0.14)
Degree of religiosity	Blackmon[75]	NS	
Personality traits	Kovach[60]	S	F(8,145) = 4.899
Self esteem<br>(n=2 studies)	Lerner[77]<br>Simpson[68]	S<br>S	- (β = -0.094)<br>- (β = -0.12)
Self efficacy<br>(n=2 studies)	Lerner[77]<br>Simpson[68] - function<br>Simpson[68] - challenges	NS<br>NS<br>S	 <br>+ (β = 0.17)
Family responsibilities	Parsons[30]	NS	
Burnout	Proenca[65]	S	- (β = -0.29)
Satisfaction with work itself	Berg[48]	S	+ r = 0.41)
Adjustment to geriatrics work	Berg[48]	S	+ (r = 0.38)
Perceived demand for physical and psychic strength	Berg[48]	NS	
Positive/negative resident relations	Albanese[46]	S	+/- (r = 0.22/ -0.21)
Experience with elderly in childhood (lack of)	Friedman[52]	S	- (β = -0.11)
Satisfaction with coworkers<br>(n=2 studies)	Blackmon[75]<br>Purk[66]	S<br>NS	+ (β = 0.44)
**5. OTHER (n=5 STUDIES)**			
Number of jobs in last 5 years	Choi[50]	NS	
Personal opportunity	Parsons[30]	S	+ (β = 0.312)
Perceived ethnic/racial bias	Ramirez[67]	S	+ (r = 0.20

Category	First Author	Significance (S = p<.05)	Direction (Magnitude)
Cultural competency	Allensworth-Davies[47]	S	+ ($\beta = 0.41$)
Respect for suggestions by people who make decisions about patient care	Friedman[52]	S	+ ($\beta = 0.22$)
Respect for choices in types of assignments	Friedman[52]	NS	

Additional File 3-2: Organizational Characteristics (reported *less* than 4 times)

Category	First Author	Significance (S = p < .05)	Direction (Magnitude)
**1. FACILITY (n=6 STUDIES)**			
Change in satisfaction with nursing home	Blackmon[75]	NS	
Company policy	House[87]	NS	
Facility ownership	Choi[50]<br>Gittell[54]	NS<br>NS	
Facility size<br>(n=3 studies)	Albanese[46]<br>Choi[50]<br>Gittell[54]	NS<br>S<br>NS	 OR = 1.43**
Location<br>(n=2 studies)	Choi[50]<br>Lerner[77]	NS<br>NS	
Occupancy rate	Albanese[46]	NS	
Proportion of Medicaid residents<br>(n=2 studies)	Albanese[46]<br>Choi[50]	S<br>NS	- (r = -0.13)
Proportion of Medicare residents<br>(n=2 studies)	Albanese[46]<br>Choi[50]	NS<br>NS	
**2. WORK ENVIRONMENT (N= 18 STUDIES)**			
Working conditions<br>Change in attitude toward the elderly<br>Chance to form close relationships with patients	House[87]<br>Blackmon[75]<br>Friedman[52]	NS<br>NS<br>NS	
High proportion of complex residents<br>Positive/negative visitor relations<br><br>Interpersonal relationship with nurse/supervisor<br>Frequency of rotation	Ramirez[67]<br>Albanese[46]<br><br>House[87]<br><br>Ramirez[67]	S<br>S<br><br>NS<br><br>NS	- (r = -0.11--0.24)<br>+/- (r = +0.30/-0.20; β = -0.13)
Working night shift (vs.day/evening)	Albanese[46]	S	- (r = -0.12)
Work related injury	Choi[50]	S	OR = 0.53**
Absenteeism	Walborn[73]	NS	
Promotion	Purk[66]	S	*
Recognition<br>(n=2 studies)	Garland[53]<br>House[87]	S<br>NS	+ (r = 0.34)
Rewards<br>(n=2 studies)	Parsons[30]<br>Thompson[71]	NS<br>S	 *
Working in PACE program	Friedman[52]	S	+ (β = 0.12)
SCU (Special Care Unit) vs. non-SCU	Ramirez[67]	NS	
Participation in team meetings<br>(n=2 studies)	Friedman[52]<br>Ramirez[67]	NS<br>S	 + (β = 0.11)
Interpersonal relationships with peers	House[87]	NS	
Work content<br>(n=3 studies)	House[87]<br>Purk[66]<br>Thompson[71]	NS<br>NS<br>NS	
Quality of care<br>(n=2 studies)	Kostiwa[59]<br>Thompson[71]	S<br>NS	+ (r = 0.43-0.63)
Receiving feedback<br><br>(n=3 studies)	Allensworth-Davies[47]<br>Friedman[52]<br>Tyler[72]	S<br>NS<br>S	*<br><br>*
Enough time	Garland[53]	S	+ (r = 0.18)

Category	First Author	Significance (S = p < .05)	Direction (Magnitude)
Others care how well you do your job	Garland[53]	S	+ (r = 0.22)
Staff presence during interventions	Goldwasser[74]	S	F(3,23) = 7.13
Variety	Friedman[52]	NS	
**3. SUPERVISION (n=12 STUDIES)**			
Immediate supervisor status	Albanese[46]	NS	
Nursing director length of employment	Albanese[46]	NS	
Nursing director professional membership	Albanese[46]	S	+ (r = 0.17)
Administrator education	Albanese[46]	NS	
Relationship with supervisor<br>(n=2 studies)	Albanese[46]<br>Berg[48]	S<br>S	-/+ (r = -0.33-0.36)<br>+ (r = 0.35)
Perception of being valued by employer	Choi[50]	S	OR = 4.15**
Supervision<br>(n=3 studies)	Garland[53]<br>Parsons[30]<br>Purk[66]	S<br>S<br>NS	+ (r = 0.48)<br>+ (β = 0.153)
Supportive supervision<br>(n=3 studies)	Choi[50]<br>McGilton[63]<br>Proenca[65]	S<br>S<br>NS	OR = 4.09**<br>+ (β = 0.36)
Supervisors	Thompson[71]	NS	
Management keeping employees informed	Parsons[30]	S	+ (β = 0.111)
Positive charge nurse behaviours	Walborn[73]	S (4/5)	+ (r = 0.205-0.270)
**4. STAFFING (n=4 STUDIES)**			
New NAs	Parmalee[64]	NS	
CNA hours per patient day	Choi[50]	S	OR = 1.30**
CNA overtime hours	Albanese[46]	S	+ (r = 0.16)
Turnover	Parsons[30]	S	- (r = -0.424)
**5. OTHER (n=10 STUDIES)**			
Exclusion	Parmalee[64]	S	- (β = -0.401)
Respect	Parmalee[64]	NS	
Social rewards	Parsons[30]	S	+ (β = 0.148)
Security	House[87]	NS	
Ongoing inservice	Friedman[52]	S	+ (β = 0.20)
Relational coordination (management of the interdependencies between employees who carry out tasks)	Gittell[54]	S	+ (r = 0.30)
Treatment condition (reminiscence vs. present focused)	Goldwasser[74]	NS	
Responsibility (given greater responsibility)	House[87]	S	NS
Status (valued by others)	House[87]	S	NS
Opportunity (to learn and develop)	Kuo[61]	S	+ (β = 0.27)
Outcome expectations for performance of restorative care activities	Lerner[77]	NS	
Observed performance of restorative activities	Lerner[77]	S	+ (β = 0.095)
Outcome expectations	Simpson[68]	NS	
Skill variety	Tyler[72]	S	*
Task identity	Tyler[72]	S	*
